# NetDI: Methodology Elucidating the Role of Power and Dynamical Brain Network Features That Underpin Word Production

**DOI:** 10.1523/ENEURO.0177-20.2020

**Published:** 2021-01-05

**Authors:** Sudha Yellapantula, Kiefer Forseth, Nitin Tandon, Behnaam Aazhang

**Affiliations:** 1 Department of Electrical and Computer Engineering, Rice University, Houston, TX 77030; 2 Department of Neurosurgery, McGovern Medical School at University of Texas Health, Houston, TX 77005

**Keywords:** directed cortical stimulation, directed information, dynamics, ECoG, graph theory, human language

## Abstract

Canonical language models describe eloquent function as the product of a series of cognitive processes, typically characterized by the independent activation profiles of focal brain regions. In contrast, more recent work has suggested that the interactions between these regions, the cortical networks of language, are critical for understanding speech production. We investigated the cortical basis of picture naming (PN) with human intracranial electrocorticography (ECoG) recordings and direct cortical stimulation (DCS), adjudicating between two competing hypotheses: are task-specific cognitive functions discretely computed within well-localized brain regions or rather by distributed networks? The time resolution of ECoG allows direct comparison of intraregional activation measures [high gamma (h*_γ_*) power] with graph theoretic measures of interregional dynamics. We developed an analysis framework, network dynamics using directed information (NetDI), using information and graph theoretic tools to reveal spatiotemporal dynamics at multiple scales: coarse, intermediate, and fine. Our analysis found novel relationships between the power profiles and network measures during the task. Furthermore, validation using DCS indicates that such network parameters combined with h_γ_ power are more predictive than h_γ_ power alone, for identifying critical language regions in the brain. NetDI reveals a high-dimensional space of network dynamics supporting cortical language function, and to account for disruptions to language function observed after neurosurgical resection, traumatic injury, and degenerative disease.

## Visual Abstract

**Figure:**
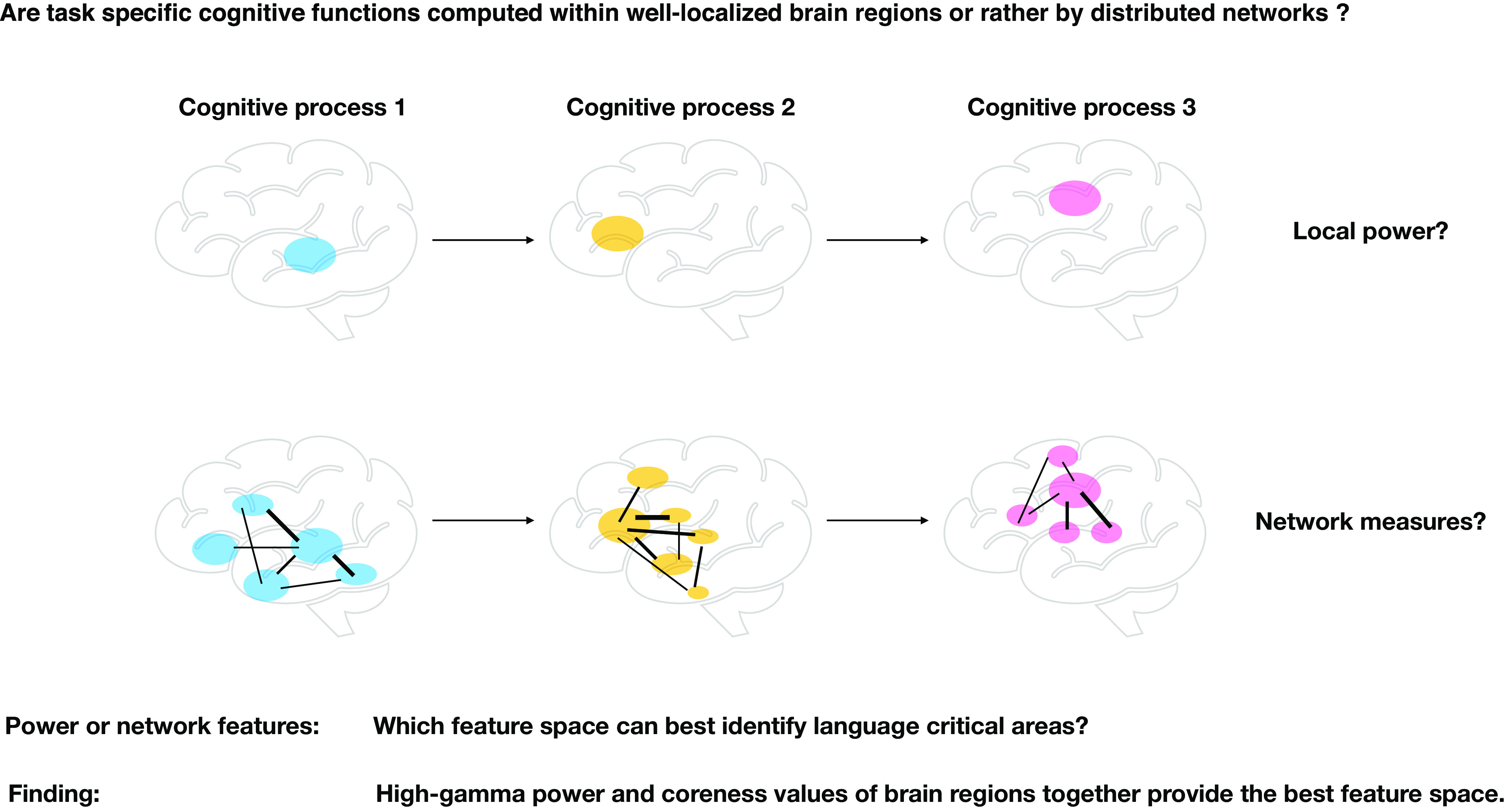

## Significance Statement

This work quantifies the network phenomena of distributed cortical substrates supporting language. First, estimated causality among brain regions was assessed with directed information (DI). Second, a graph theoretic framework extracted task related dynamics from the causal estimates. Finally, we validated these functionally defined networks against the gold standard for causal inference, behavioral disruption with direct cortical stimulation (DCS). We demonstrate that the network measures combined with power have greater predictive capability for identifying critical language regions than discrete, regional power analyses alone.

## Introduction

Historically, language has been studied in a localized manner, to attribute specific roles to individual neural substrates. This perspective is evidenced by activity in distinct brain regions measured by the blood-oxygen level-dependent responses of functional MRI (fMRI; Price, 2010) or by high-γ (h_γ_) power (>60 Hz) in electrocorticography (ECoG) recordings (Crone et al., 2001; Edwards et al., 2005; Towle et al., 2008; Cogan et al., 2014; Conner et al., 2014; Flinker et al., 2015; Riès et al., 2017). Furthermore, lesion studies have demonstrated that different brain lesions separably impair discrete aspects of the language system (Geschwind, 1974; Hickok and Poeppel, 2007). More recently, it is becoming obvious that linguistic processes are better characterized as network phenomena (Fedorenko and Thompson-Schill, 2014; Braun et al., 2015; Medaglia et al., 2015; Bassett et al., 2015; Blank et al., 2016; Domenico, 2017; Herbet and Duffau, 2020), as it has been theorized that network properties better explain the complex and transient dynamics during linguistic cognition (Chai et al., 2016; Herbet and Duffau, 2020; Salehi et al., 2020). We quantified network dynamics during a word generation task to evaluate the hypothesis that linguistic operations engaged during picture naming (PN) are better explained by including the network properties and local activity of specific cortical loci than the activity at each locus itself. Further, we probe whether the critical nature of these sites, evidenced by disruption by direct stimulation is related to their centrality measures within the language network.

Intracranial electrodes in humans provide a unique opportunity to resolve this central debate by enabling direct recordings of neural processes with sufficient temporal resolution and spatial specificity to resolve transient network dynamics. Furthermore, these electrodes can be used to induce targeted transient dysfunction via electrical stimulation, providing causal functional inference. We used both modalities to directly compare regional activation measures (h_γ_ power) with network measures.

**Figure 1.**
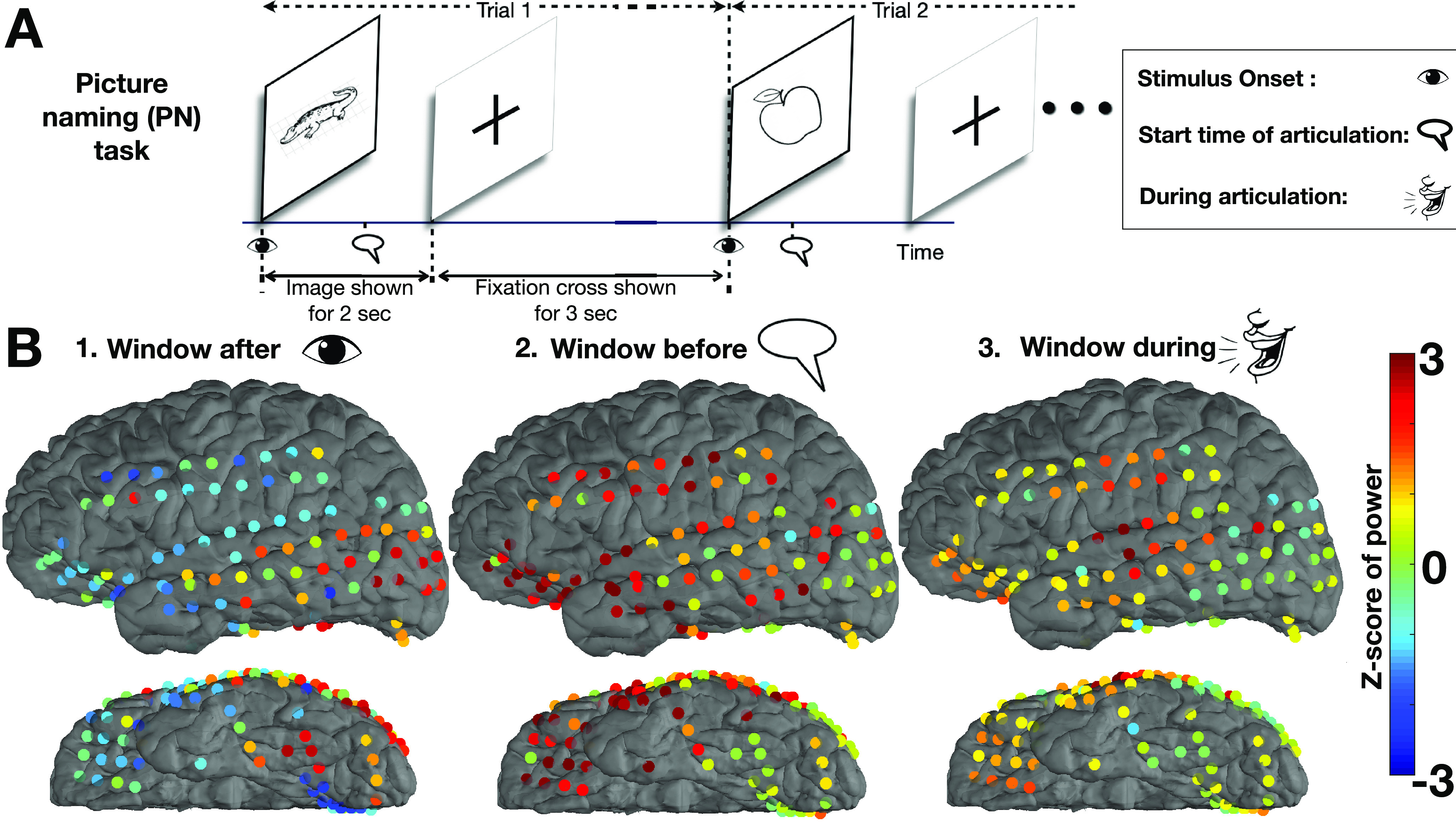
***A***, Multiple (>200) trials of the PN task were performed. For each trial, an image from the Boston naming test (Kaplan et al., 1983) was shown; patient articulated when the image was identified. ***B***, The *z* scores of mean h*_γ_* power responses across trials are shown for a patient in three time windows. ***B1***, In the window 200–456 ms relative to stimulus onset, increased power in visual cortex and decreased power in frontal regions compared with the baseline are observed. ***B2***, The prearticulation window has multiple electrodes with an increase in h*_γ_* power in frontal, motor, and temporal regions. ***B3***, The postarticulation window has increase in power in the auditory cortex, aligning with the task wherein the patient hears themselves speak.

ECoG during PN (Fig. 1*A*) was recorded from subdural grid electrodes implanted in the left language dominant hemisphere of seven patients. We developed a holistic framework, network dynamics using directed information (NetDI), which extracts time-varying network dynamics using information and graph theoretic tools.

DI (Massey, 1990; Kramer, 1998) was used to measure directional information flow between time-series across brain regions. DI measures are valuable in neuroscience (Quinn et al., 2012; Malladi et al., 2016; Murin et al., 2016) given its broad applicability to a wide range of electrophysiological recordings, without model assumptions. Traditional causality metrics like Granger causality (GC; Seth et al., 2015) rely on data belonging to linear auto-regressive models, which ignores nonlinear relationships among brain signals (Kowalik et al., 1996; Stokes and Purdon, 2017). Furthermore, DI is equivalent to GC (Amblard and Michel, 2011, 2012) when the data are truly linear and Gaussian, and it is closely related to Transfer Entropy (Schreiber, 2000; Liu, 2012; Liu and Aviyente, 2012), under the Markov condition. The causality yielded by DI (in bits) is causal in the Wiener–Granger sense (Bressler and Seth, 2011); crucially, no assumptions are imposed on the underlying probability distribution of the data.

This work proceeds in three stages. First, we measured causality between brain regions and extracted task related dynamics from the calculated causality values, to resolve network measures at coarse, intermediate, and fine scales. Second, we evaluated the relationship between network measures and the local power responses. We analyzed the relationships between node centrality, given by “coreness of nodes,” which prior work has identified as a good measure of a node’s influence in the network (Kitsak et al., 2010; Pei et al., 2014; Shin et al., 2016) and h*_γ_* power. We also looked at relationships between fine scale measures, in-degrees, out-degrees, and power. These node metrics are the components behind the node centrality measure and throw light on how they influence the node centrality. Third, we evaluated the relationship between whether or not stimulation at a given cortical site disrupted language and its corresponding network measures as estimated by NetDI. Various feature spaces were compared, power feature, network features and combined power and network feature spaces, to examine which feature space has the best discriminability between language positive and language negative areas, based on ground truth data given by clinical functional mapping by direct cortical stimulation (DCS).

## Materials and Methods

### Picture naming task

Patients performed a PN task, where they were shown images from the Boston naming test (Kaplan et al., 1983). Each trial consisted of an image being displayed on a screen for 2 s, followed by a fixation cross for at three more seconds. Multiple (>200) trials of the PN task were performed for each patient. For each trial, an image from the Boston naming test was shown; patient articulated when the image was identified (details in Table 1). Figure 1*A* illustrates the experimental methodology.

### Data and preprocessing

Intracranial electroencephalography (iEEG) data were obtained from subdural grid electrodes implanted in left hemisphere in patients before resective surgery for intractable epilepsy. Electrodes that had close proximity to the sites of seizure onset, interictal spikes or had >10-dB noise in the 60-Hz band, were considered to be bad channels, and were excluded from the analysis. Also, data from bad trials across all channels were excluded, if the trials included epileptiform activity, or had technical errors. The exclusion of bad electrodes and bad trials were done similar to Conner et al. (2014) and Kadipasaoglu et al. (2014, 2015). Seven patients were analyzed and details of the number of electrodes and the number of trials used for analysis are shown in Table 1, along with patient demographics. iEEG data were preprocessed by first performing a common average reference, where the electrodes within each subject were re-referenced by subtracting the common average of all electrodes (Kadipasaoglu et al., 2017); 60-Hz and higher harmonics were removed using bandstop IIR butterworth filters of order 6. Zero phase filtering was also performed, to ensure that the features in the filtered waveforms were preserved exactly at the same time locations as the unfiltered signals.

**Table 1:** Patient information

Patient #	Age(gender)	# Electrodes	# trials	Grid side
1	42(M)	136	262	Left
2	23(M)	89	264	Left
3	34(F)	91	275	Left
4	23(M)	107	206	Left
5	45(F)	112	223	Left
6	51(F)	85	203	Left
7	24(M)	91	250	Left

### Analysis time windows

**Figure 2.**
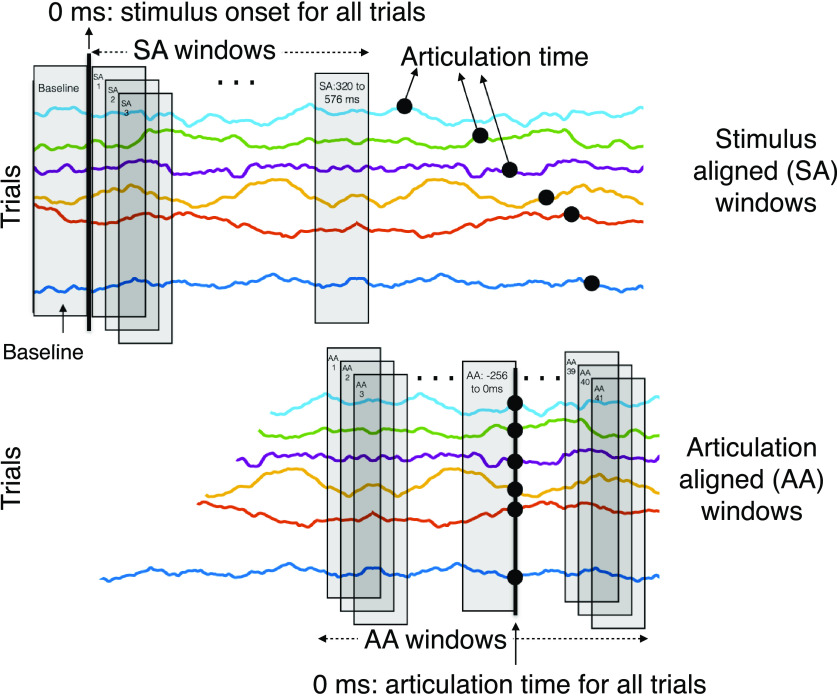
SA windows are aligned to stimulus onset, AA to onset of articulation. The last SA window did not overlap with the first AA window, to ensure temporal continuity. 11 SA (start times 0–320 ms, sliding by 32 ms) and 41 AA (start times −480–768 ms, sliding by 32 ms) windows were used in analysis.

“Stimulus onset” refers to the time at which the picture came on screen, and “articulation time” corresponds to the speech onset time for the verbal response. Data analysis was done in 256-ms windows, and the window before stimulus onset was chosen as the baseline. Windows were called stimulus aligned (SA) or articulation aligned (AA) based on whether the trial data in the window was aligned to the stimulus onset or articulation time, respectively. Windows are denoted as SA/AA: <start time > to <end time > in this article (Fig. 2). For example, AA: −256 to 0 ms represents a window that starts 256 ms before articulation. The 0-ms time has a dual meaning based on the context. It is used to represent stimulus onset in SA windows and articulation time in AA windows. All analyses were done in *W *=* *52 windows, consisting of 11 SA (start times 0–320 ms, sliding by 32 ms) and 41 AA (start times −480 to 768 ms, sliding by 32 ms) windows.

### Power analysis

After data pre-processing, the time series from all electrodes were filtered into the h*_γ_* band (60–150 Hz) using a zero phase, order 6, IIR butterworth filter, to obtain a h*_γ_* signal. In a given SA or AA window, let us denote the raw time-series as $\left\{X_{1},X_{2},...,X_{N}\right\}$. The h*_γ_* signal represented as $\left\{g_{1},g_{2},...,g_{N}\right\}$, was obtained after filtering the raw time-series, where *N* is the length of the h*_γ_* time-series in that window (*N *=* *256, since sampling frequency was 1 kHz). For each electrode’s recordings, power was calculated in moving SA time windows over the trial duration, to form a h*_γ_* power time series for each trial. The instantaneous power series of h*_γ_* power in window *w*, in a given trial is given by $\left\{g_{1}^{2},g_{2}^{2},...,g_{N}^{2}\right\}$. The trial-averaged instantaneous power in window *w* is $G_{w}(i)=\{E[g_{1}^{2}],E[g_{2}^{2}],...,E[g_{N}^{2}]\}$, where i=1,2. . . ,N and E[.] is the mean across the trials. The trial-averaged power series $G_{w}$ in window *w* were converted into *z* scores $Z_{\text{h}\gamma }(w)$, using the mean and SD of the power in the baseline window *w_b_*. This resulted in normalized *z* score power series for SA data. Similarly, *z* score of power was calculated for AA data. To have responses indexed to both stimulus-driven and articulation-related processes, we used a subset of both SA and AA windows, based on whether we were analyzing data right after the stimulus onset, or around articulation, respectively. Remark: Figure 1 shows power normalized to the trial window (not baseline) solely for visualization purposes, to clearly discern the temporal location of the normalized power responses. For all data analysis involving power, the power was normalized using the baseline window, as described in Equation 1.

(1) $$\begin{array}{l} Z_{\text{h}\gamma }(w)=\frac{E[G_{w}]-E[G_{w_{b}}]}{\sigma [G_{w_{b}}]} \end{array},$$

where this E[.] is the time-average of the power series $G_{w}(i)$ in the window, $E[G_{w_{b}}]$ is the mean power in the baseline window *w_b_*, and $\sigma [G_{w_{b}}]$ is the SD of the baseline power.

### Directed Information

DI was estimated in a model free manner using the computationally efficient Kraskov, Stögbauer, and Grassberger (KSG) estimator (Kraskov et al., 2004; Gao et al., 2018), which is based on a k-nearest neighbors (kNN) approach to measuring mutual information (MI). DI was estimated between every pair of channels in each SA and AA time window in a data-driven manner. Every trial was considered to be an independent sample path of an unknown underlying random process, and pairwise DI was estimated using all the trials in a given window. Given two raw time series from a pair of electrodes $X_{1}^{N}=\{X_{1},X_{2},...,X_{N}\}$ and $Y_{1}^{N}=\{Y_{1},Y_{2},...,Y_{N}\}$, where $X_{i},Y_{i}\in R$, DI from $X_{1}^{N}$ to $Y_{1}^{N}$ is denoted as $I(X_{1}^{N}\to Y_{1}^{N})$, and is defined as the following:

(2) $$\begin{array}{l} I(X_{1}^{N}\to Y_{1}^{N})=\sum_{i=1}^{N}I(X_{1}^{i};Y_{i}|Y_{1}^{i-1})\\ =\sum_{i=1}^{N}\left(h(X_{1}^{i}|Y_{1}^{i-1})+h(Y_{i}|Y_{1}^{i-1})-h(X_{i}^{i},Y_{i}|Y_{1}^{i-1})\right) \end{array},$$

where the right hand side of Equation 2 is the conditional MI between time series $X_{1}^{i}$ and single sample point *Y_i_*, conditioned on the past *i* – 1 samples $Y_{1}^{i-1}$. DI can also be expressed as sums of conditional differential entropies given by *h*’s in Equation 2. By definition, differential entropy *h*(*Z*) of a continuous random variable *Z* with a probability density function *f*(*z*) is the following:

(3) $$\begin{array}{l} h(Z)=-\int_{S}f(z)\log f(z)dz\;\;\;\;\;\text{(where S is the support of the random variable }Z). \end{array}$$

Also, a conditional differential entropy term can be expressed as a difference of two differential entropies:

(4) $$\begin{array}{l} h(Z|W)=-\int f(z,w)\log f(z|w)\;dz\;dw\\ h(Z|W)=h(Z,W)-h(W) \end{array}.$$

From Equations 2, 4, DI can be expanded as the following:

(5) $$I(X_{1}^{N}\to Y_{1}^{N})=\sum_{i=1}^{N}\left(h(X_{1}^{i},Y_{1}^{i-1})+h(Y_{i},Y_{1}^{i-1})-h(Y_{1}^{i-1})-h(X_{1}^{i},Y_{i},Y_{1}^{i-1})\right).$$

Each entropy term in Equation 5 was estimated using the KSG estimator (Kraskov et al., 2004), which uses a kNN approach, similar to the methodology described in Murin (2017). The implementation of DI was written in MATLAB using the kNN tools from Trentool (Lindner, 2011; Lindner et al., 2011). ECoG data were assumed to be Markovian of order *m*, i.e., samples only depend on the past *m* samples. Based on a non-parametric method of estimating memory order for ECoG (Murin et al., 2019), and from other similar work (Malladi et al., 2016; Murin et al., 2016), a memory order of 150 ms was used, achieved by using downsamples of the data for estimation. The final equation used for estimation of DI rate $\hat{I}(X_{1}^{\tilde{N}}\to Y_{1}^{\tilde{N}})$ is given by:

$$\hat{I}(X_{1}^{\tilde{N}}\to Y_{1}^{\tilde{N}})=\frac{1}{\tilde{N}-m}\sum_{i=m+1}^{\tilde{N}}[\hat{h}\left({\tilde{X}}_{i-m+1}^{i},{\tilde{Y}}_{i-m}^{i-1}\right)+$$

(6) $$\hat{h}\left({\tilde{Y}}_{i},{\tilde{Y}}_{i-m}^{i-1}\right)-\hat{h}\left({\hat{Y}}_{i-m}^{i-1}\right)-\hat{h}\left({\tilde{X}}_{i-m+1}^{i},{\tilde{Y}}_{i},{\tilde{Y}}_{i-m}^{i-1}\right)],$$

where *m* is the number of past samples, $\tilde{X}$ and $\tilde{Y}$ are the downsampled versions of *X* and *Y*, respectively, $\hat{h}$’ s are the estimated differential entropies, and $\tilde{N}$ is the length of the downsampled signal.

### Bootstrapping and bias-correction of DI estimate

The bias of the empirical estimation of DI is defined as the difference between the true value of DI and the estimated value of DI. All estimators incur bias because of the amount of data samples being finite. The KSG estimator is known to have a negative bias for small sample sizes (Kraskov et al., 2004). To allow for comparisons of DI values, bias-correction was performed for every DI estimate, analogous to debiasing in GC literature (Barrett et al., 2012; Barnett and Seth, 2014). Bias-correction was performed by generating multiple samples of “zero DI” under the null hypothesis by multiple time shuffles of each trial, of one of the channels *X*, similar to Diks and DeGoede (2001) and Malladi et al. (2016). This ensured that all temporal dependencies were removed between the two channels, and the estimated null DI was denoted as ${\hat{I}}_{\emptyset }(X_{1}^{N}\to Y_{1}^{N})$. The average null DI estimate was subtracted from the original estimated DI, to obtain the bias-corrected estimate of the information flow from X to Y, denoted as the following:

(7) $${\tilde{I}}_{w}(X_{1}^{N}\to Y_{1}^{N})=\hat{I}(X_{1}^{N}\to Y_{1}^{N})-E\left[{\hat{I}}_{\emptyset }((X_{1}^{N}\to Y_{1}^{N})\right],$$

where E is the expectation operator. Henceforth, all pairwise DI values under discussion refer to bias corrected values, denoted by ${\tilde{I}}_{w}(X_{1}^{N}\to Y_{1}^{N})$ to represent information flow from channel *X* to channel *Y*, in time window *w*.

### Multiscale graph theoretic framework

**Figure 3.**
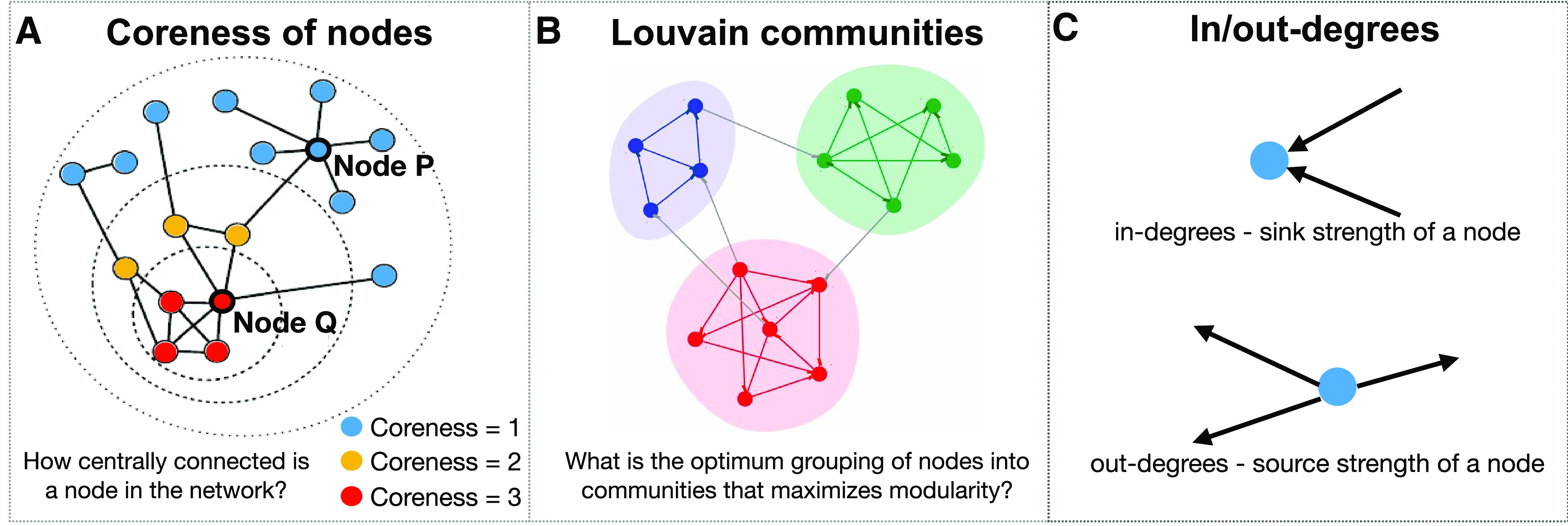
***A***, Coreness of a node is a measure of the node’s centrality in the network. While typically, a higher coreness value corresponds to a higher node degree, it is not always the case. For example, both nodes P and Q have a degree of 6, yet node P has a coreness value of 1, while node Q has a coreness value of 3. ***B***, Louvain communities. ***C***, Fine scale network measures given by in-degrees and out-degrees.

**Figure 4.**
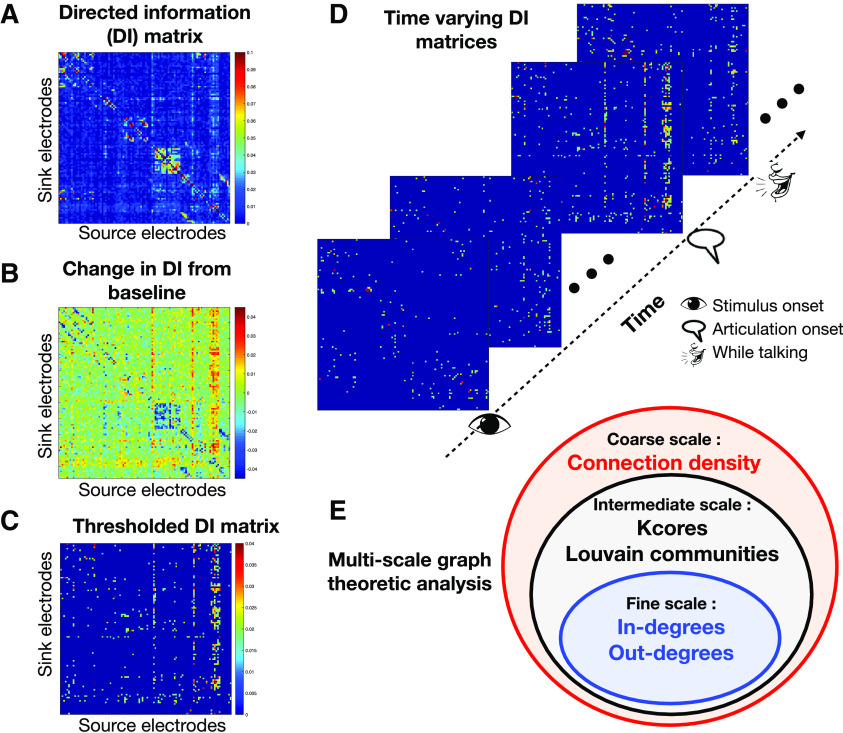
***A***, DI matrix in a window; *x*- and *y*-axes are the electrodes and the ${\left(i,j\right)}^{\text{th}}$ element represents DI from the $j^{\text{th}}$ to the $i^{\text{th}}$ electrode. ***B***, The DI matrix relative to the baseline (“change in DI” matrix) is obtained by subtracting the baseline DI matrix from the DI matrix of that window. ***C***, The thresholded DI matrix, which only retains the top positive increases in DI, based on a threshold value T (described in Materials and Methods). ***D***, *W *=* *52 “increase in DI” matrices were generated per patient. ***E***, Multiscale graph analysis.

A graph theoretic framework (Bullmore and Sporns, 2009, 2012; Sporns, 2010) was used to evaluate connectivity between brain regions. We construct a graph *G_w_* = (*V*,*E*) in time window *w*, where *V* is the set of *M* nodes in the graph that represent brain electrodes, *E* is the set of edges that denote connections between the nodes. The ${\left(u,v\right)}^{\text{th}}$ element of the adjacency matrix $a_{w}(u,v)$ is given by the directed edge metric from node *u* to node *v*, defined as the change in DI from the baseline window. Thus, $a_{w}(u,v)={\tilde{I}}_{w}(u_{1}^{N}\to v_{1}^{N})-{\tilde{I}}_{b}(u_{1}^{N}\to v_{1}^{N})$, where *b* represents the baseline window and *N* the length of the time window. The edge metric captures the “change in DI from baseline” and represents the changes occurring because of the task dynamics. For each of the seven patients, a total of *W*(=52) brain graphs were obtained from 52 time windows (11 SA and 41 AA windows). To control for spurious edges, the adjacency matrices were thresholded using a combination of density thresholding and global thresholding techniques (van Wijk et al., 2010). Density thresholding for an adjacency matrix is a technique where the threshold is chosen such that the resulting network has a certain density of edges. Global thresholding for a group of graphs uses a single threshold value (T), and retains all the edges greater than the common threshold. In this work, density thresholding is done in one time window, to determine what T should be, and is then applied globally for all the time windows. Density thresholding was done on a graph corresponding to the window before articulation (AA: –256 to 0 ms), by retaining only the top 5% of the positive increases in DI values, among all pairs of nodes. The cutoff value determined the patient-specific threshold (*T*), which was then globally applied to all the remaining *W* – 1 windows. The window AA: –256 to 0 ms was chosen because of its high density of connections, to be very stringent and have a high value of threshold *T*. The results were found to be largely independent of the window chosen for density thresholding, as using a different window only changed the value of *T* slightly, which affected a few individual connections, but it was not sufficient to change the subsequent graph theoretical metrics. After thresholding, the elements of the adjacency matrix *A_w_*(*u*,*v*) for window *w* are given by

(8) $$A_{w}(u,v)=\{\begin{array}{ll} a_{w}(u,v) & \text{if }a_{w}(u,v)\geq T\\ 0 & \text{if }a_{w}(u,v)<T \end{array},$$

and the edges now represent “increase in DI from baseline.” This thresholding technique retains values of DI that are much greater than baseline, and discards “decreases in DI from baseline.” This makes the adjacency matrices positive, allowing for the use and interpretation of most graph processing techniques. The final results are carefully interpreted within the framework of networks built out of increased information flow among brain regions. For each patient, the resulting time varying graphs were analyzed using a multiscale analysis procedure (Fig. 3) using metrics that shed light on the underlying process at various scales, coarse, intermediate, and fine (Fig. 4).

#### Coarse scale, connection density

**Figure 5.**
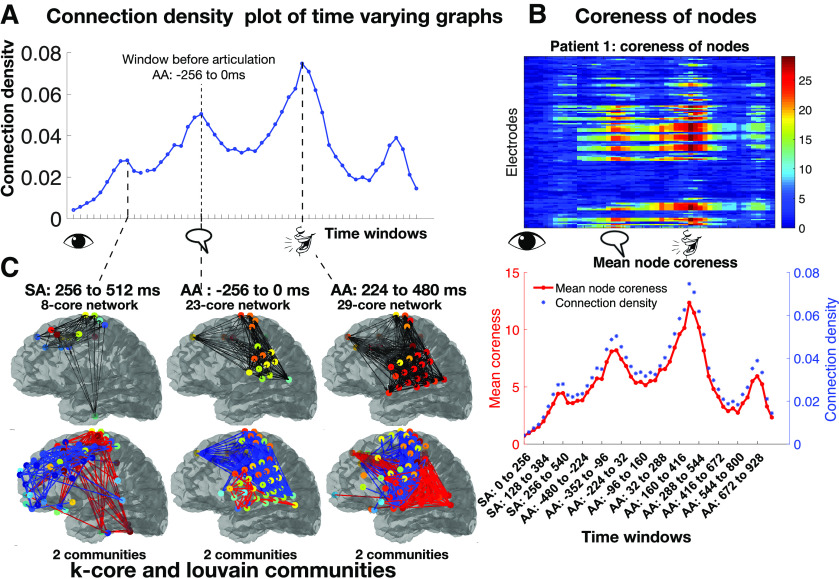
***A***, Connection densities vary smoothly across time windows, with a local maximum occurring before articulation. ***B***, Coreness of nodes heatmap identifies sets of nodes in the brain related to changes in connection density. The average coreness across nodes (shown in red below the heatmap), provides the same information as the coarse scale metric (in blue). ***C***, The first row shows the maximal k-core network in windows where peaks were found in the coarse metric. Connections are shown in black lines. The max k-core network is a very strongly interconnected core of the graph. The second row shows the results of the Louvain analysis. The colors of directed lines only indicate that the nodes belong to the same community. Significant communities, Bonferroni corrected *p *<* *0.05 are shown.

A patient with *M* electrodes has *W* thresholded adjacency matrices *A_w_*, for each window *w*, of size *M* × *M*. The connection density of each matrix defined as the ratio of the number of connections (non-zero values) in the adjacency matrix, to the total number of possible connections *M*^2^-*M*. The results of one patient’s connection density versus *W* time windows is shown in Figure 5*A*.

#### Intermediate scale, coreness of nodes

K-cores analysis (Hagmann et al., 2008; Modha and Singh, 2010) is an intermediate scale graph theoretic metric, calculated using directed binarized graphs (Rubinov and Sporns, 2010a,b). K-cores of a graph are a set of connected components that remain, after all vertices of degree less than k have been removed, in an iterative manner. Coreness of node quantifies the highest k-core network a given node belongs to (Fig. 3*A*). The coreness values of the nodes were evaluated as follows (Shin et al., 2016): First, the 1-core network was identified by finding the isolated 0-degree nodes of the graph. These nodes were given a coreness value of 0, and then deleted from the network to reveal the 1-core network. Next, the 2-core network was identified, and the nodes deleted at this step were given the coreness value of 1. This process was repeated until every node was given a coreness value, until the largest k-core subnetwork of each graph *G_w_* was found. The coreness of all nodes of a patient versus time windows has been plotted as a heatmap, as shown in Figure 5*B*. This revealed which set of nodes were involved in highly connected subnetworks, and in which time windows this occurred.

#### Intermediate scale, Louvain algorithm: maximizing modularity

The Louvain algorithm (Reichardt and Bornholdt, 2006; Blondel et al., 2008; Ronhovde and Nussinov, 2009; Sun et al., 2009) is a fast, heuristic, agglomerative community detection algorithm, that finds the optimal partition structure of the nodes into communities, by maximizing a measure of partition quality; the modularity index *Q* (Newman and Girvan, 2004), example in Figure 3*B*. The Louvain algorithm in this work used an adapted modularity index suitable for directed weighted networks (Rubinov and Sporns, 2010a,b, 2011). It uses a greedy optimization phase which randomly selects the starting node, leads to an inherent variability of the Louvain algorithm. To overcome this, consensus clustering was done (Rubinov and Sporns, 2011; Lancichinetti and Fortunato, 2012; Dwyer et al., 2014), where the algorithm was run *R* = 100 times, and an *R* × *R* module allegiance matrix (Bassett et al., 2013, 2015) was created. The community detection algorithm was then run for a second time on this module allegiance matrix, which revealed the most robust partition of the data (Lancichinetti and Fortunato, 2012). The number of communities in each graph was determined by the output of the algorithm. The Louvain communities in three windows for a patient are shown in Figure 5*C*.

#### Fine scale, in-degrees and out-degrees of nodes

**Figure 6.**
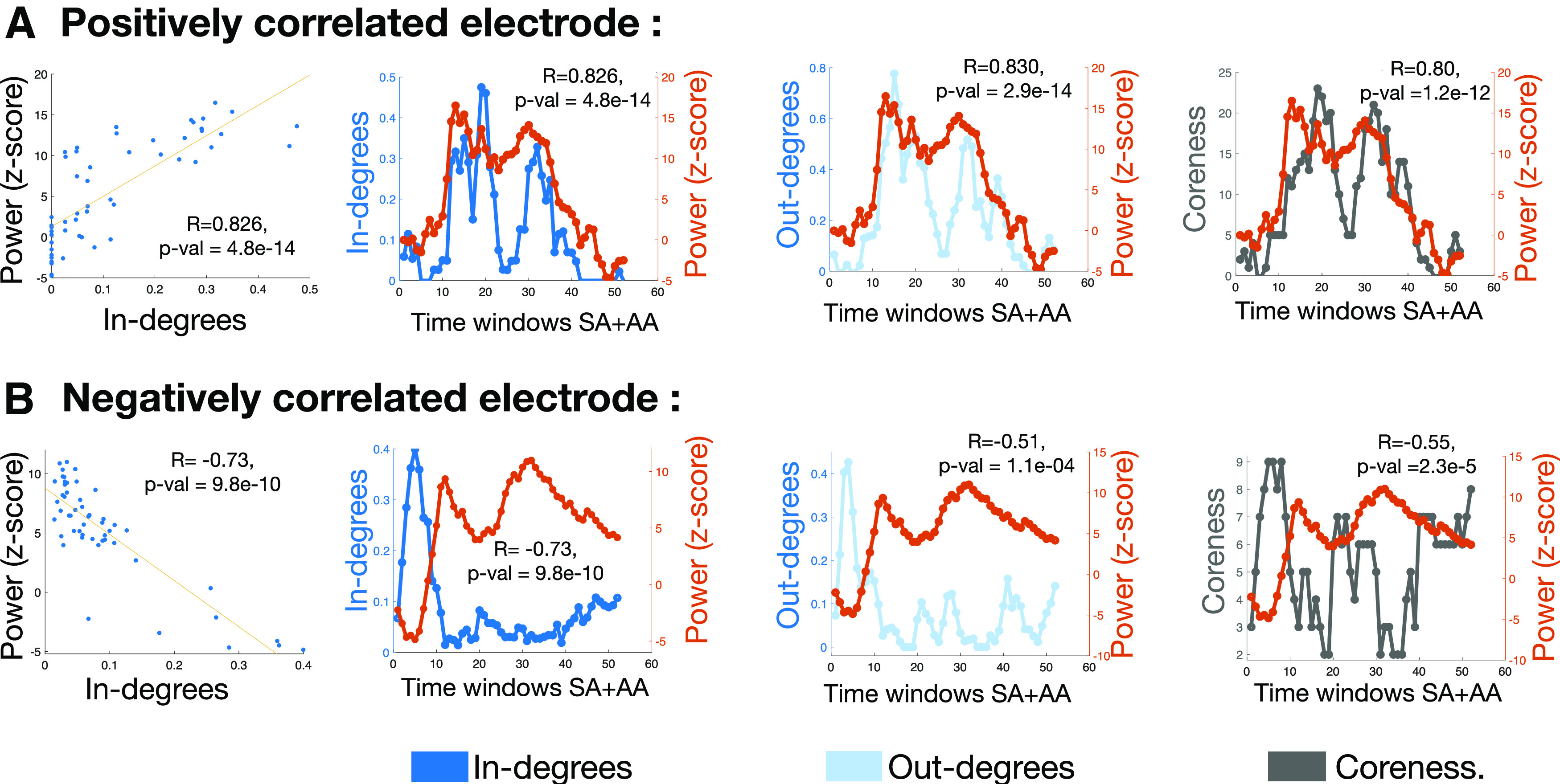
***A***, An inf-frontal gyrus pars opercularis electrode from patient 1, that shows positive correlation between h*_γ_* power and the network features. ***B***, A negatively correlated orbital frontal cortex from patient 6. The coreness of nodes can be seen as a combined effect of in and out degrees. A detailed figure that shows the correlation coefficient of in/out degrees with other frequency bands is shown in Extended Data Figures 6-1, 6-2. A pictorial understanding of how in/out degrees correlates with power is shown in Extended Data Figure 6-3. *R* represents the correlation coefficient.

**Figure 7.**
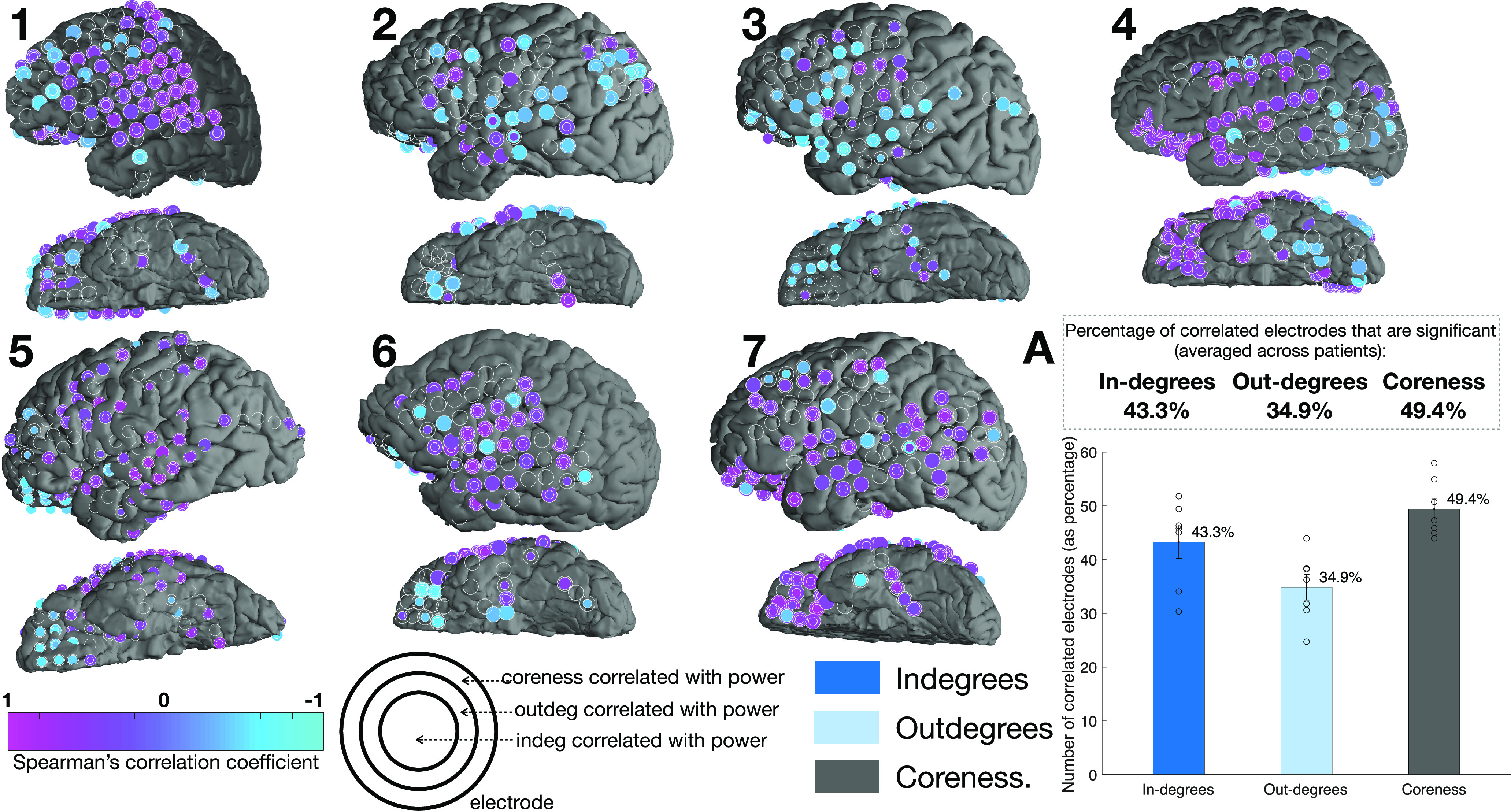
Every three-ring electrode on the brain denotes the correlation coefficient value of three feature spaces with power. The significant correlation coefficient of in-degrees and h*_γ_* power time-series are shown in the innermost circle, the correlation coefficient of out-degrees with power is denoted by the color of the middle ring, while the outer ring for each electrode’s color denotes the correlation coefficient of coreness of nodes with power. The absence of color in the outer ring, or the absence of either the middle or inner ring, denotes the lack of significant correlation in that electrode, with that feature space. This figure denotes the correlation coefficient calculated using the entire time-series, SA and AA time windows considered separately are shown in Extended Data Figure 7-1. The bar plot to the right shows the average percentage of electrodes that showed significant correlation for each feature space, after correcting for multiple comparisons (FDR, *p* < 0.05 for each feature space, per patient). It can be noted that most electrodes’ feature spaces show correlation in the same direction. Across patients, more electrodes have significant correlation of coreness of nodes feature space with power, than the other two feature spaces.

For a node v∈V, in-degrees ${\text{In}}_{w}(v)$ and out-degrees ${\text{Out}}_{w}(v)$ provide a fine grained view of the graph structure, for time windows w=1,2,. . .,W. For weighted, directed networks, in-degrees of a node is defined as the sum of the weights of the edges entering that node. ${\text{In}}_{w}(v)=\sum_{u\in V}A_{w}(v,u)$. Similarly, out-degrees of a node is defined as the sum of the weights of the edges leaving that node. ${\text{Out}}_{w}(v)=\sum_{u\in V}A_{w}(u,v)$. In-degrees is a measure of the sink strength of the nodes, while out-degrees measures source strength (Fig. 3*C*). The significant correlations between in-degrees and power, and out-degrees and power for all patients are shown in Figure 6 and Figure 7.

### Relationship between network features and power

The relationship between network features (coreness of nodes, in-degrees, and out-degrees) and power of each was evaluated to understand how network features and power could be related. We conducted the following analyses, which are summarized in the results. We analyzed positive and negative correlations of network features and h_γ_ power, results shown in Figure 6. We evaluated correlations between in-degrees, out-degrees with power in five frequency bands (h*_γ_*, γ, β, α, and θ), and demonstrated the relationships between the frequencies and the network features, results shown in Extended Data Figures 6-1, 6-2. An intuitive understanding of how in/out degrees correlate with power is shown in Extended Data Figure 6-3. Evaluated correlations between coreness of nodes, in-degrees, out-degrees with h_γ_ power, for all time windows (SA + AA; results in Fig. 7). Evaluated correlations between coreness of nodes, in-degrees, out-degrees with h_γ_ power, for time windows SA and AA separately, since the processes underlying SA and AA windows could be different (results in Extended Data Fig. 7-1).

With an understanding of how network features are related to power, we then evaluate whether network features provide additional information to the language process, compared with power alone, using data from DCS.

### Direct cortical stimulation

DCS was used to map brain function before surgeries, wherein targeted transient dysfunction was induced via electrical stimulation, providing causal functional inference. DCS revealed which node-pairs were PN positive and language negative for brain regions, in the following manner. In order to map brain function using DCS, a patient performed three tasks: a PN task, an auditory repetition task, and an auditory naming task. During each task, a pair of electrodes (node-pair) were stimulated with an electric current. If the stimulation disrupted the PN task, then that node-pair was considered PN positive. Each task was tested separately. If the current stimulation did not disrupt any of the three language tasks tested individually, then that node-pair was considered to be language negative. DCS caused a reversible temporary lesion, and allowed the doctor to assess the importance of that node-pair for brain resection. Note: The PN task for DCS is different from the PN task done for analysis using NetDI for research purposes.

### Comparison of network/power features using classification of node-pairs

The DCS data labels: PN positive or language negative for node-pairs were considered as ground truth. Using standard machine-learning classifiers and a training and testing paradigm, the accuracy of classification of node-pairs were compared using various power and network feature spaces, namely, (1) h*_γ_* power of the node-pairs, (2) in-degrees, (3) out-degrees, (4) both in-out degrees (5) coreness of nodes; and using a combined network and power feature space: (6) in-degrees and power features, (7) out-degrees and power, (8) combined in/out degrees and power feature space, and (9) combined coreness of nodes and power. Multiple classifiers were used to eliminate bias in the results because of a particular classifier. A comparison of classification accuracy across these nine feature spaces would reveal insight into which feature space had higher discriminability to classify between the PN positive and language negative node-pairs. The total number of labeled node-pairs (n), and the length of each feature space (p) together form an n × p matrix, (n < p), which is used for classification. For example, the power feature space was created by taking the power time series (from 52 windows) for each node of the node-pair, concatenating them to produce a vector of length 104 for that node-pair. Every DCS node-pair provided two data samples for classification, as features from node-pairs could be concatenated in two ways. Specifically, each DCS node-pair was used to generated another labeled node-pair by reversing the order of the nodes in which the features were concatenated, thus doubling our available labeled node-pairs. Thus, in this work, the effective n for classification varied between 26 and 46 for the patients, while p varied between 104 and 312, based on the feature space considered. The number of labeled node-pairs were sufficient to estimate classification accuracies with a 95% confidence interval. For each feature space, 5-fold cross validation was performed, with repeated random splits of the data (Varoquaux et al., 2017), keeping the training and test sets stratified, to have a balanced split among the two classes. The results are averaged over the test sets. Remark: Our previous efforts with using the original DCS node-pairs had insufficient samples for 5-fold cross validation and confidence interval estimation. Classification accuracies found using the leave-one-out cross-validation methodology were similar to the results and trends among feature spaces presented in this paper, but did not have additional statistics provided here.

A brief note on notation: TP(FP) stands for true(false) positives, TN(FN) for true(false) negatives. True positive rate (TPR), also known as sensitivity or recall is given by $\text{TPR}=\frac{\text{TP}}{\text{TP}+\text{FN}}$. True negative rate (TNR) is also called specificity or selectivity is $\text{TNR}=\frac{\text{TN}}{\text{TN}+\text{FP}}$. Precision is given by $\frac{\text{TP}}{\text{TP}+\text{FP}}$. For all patients, the number of PN positive and language negative node-pairs were not equal, so the balanced accuracy metric was used, by normalizing true positive and true negative predictions. Balanced accuracy $=\frac{\text{TPR}+\text{TNR}}{2}$.

### Statistical analysis

Significance of network-based measures were evaluated using controls and statistical tests. The statistical significance of the Louvain communities in each graph was calculated using non-parametric permutation testing (Park et al., 2009) by randomly permuting the community labels assigned to the nodes 5000 times. The final communities reported were Bonferroni corrected *p* < 0.05. The network measures in-degrees, out-degrees, and coreness of nodes were correlated with the power responses, the significant correlations, probability p < 0.05, FDR corrected were reported. All comparisons of classification accuracy among different feature spaces were reported with statistical significance for *p *<* *0.05, using *t* tests, also FDR corrected for multiple comparisons, among feature spaces.

### Code and software

The NetDI analysis was performed in MATLAB 2014b, while the DCS classification analysis was done using Python’s Sci-kit learn machine learning toolbox (Pedregosa et al., 2011) in Jupyter notebooks. Python version 3.7 was used. Code used to develop the NetDI analysis and the classification analysis based on DCS are provided online at https://github.com/ysudha/NetDI.

## Results

### Multiscale network analysis

The brain processes span multiple resolutions, and hence it was important to analyze the dynamics at multiple scales. The first step of NetDI was to estimate causal information flow between brain regions. Pairwise bias-corrected DI (see Materials and Methods) between all pairs of electrodes were calculated for each window w=1,2...,W (Fig. 4*A*), followed by a baseline normalization of DI (Fig. 4*B*) and thresholding (Fig. 4*C*). Each resulting DI matrix was interpreted as an adjacency matrix of a graph, whose nodes are fixed electrodes, and the edges between the nodes are based on the increase in DI between them. The second step of NetDI was to obtain spatiotemporal dynamics from the resulting time-series of graphs. Graph theoretic tools at coarse, intermediate, and fine scales of resolutions (Fig. 3) were then used to analyze the graphs.

Connection density is a coarse scale metric, that provided a single number per graph; the plot for patient 1 is shown in Figure 5*A*. The connection density plot had a temporal pattern, with local temporal maxima (peaks). One peak occurred exactly at the window immediately preceding articulation (AA: –256 to 0 ms). The timing of the peak suggests that the connections in that window represent “local decisions” being made. By extension, the peak in the SA windows at SA: 256 to 512 ms could relate to word identification, and the peak after articulation at AA: 224 to 480 ms may relate to a brain process that occurs while the patient is still speaking.

Coreness of nodes (Hagmann et al., 2008) is an intermediate scale metric, that was plotted as a heatmap in Figure 5*B*. It spatially identified the nodes that belonged to the higher cores, and were thus more central to the network, quantified by their coreness values. The Louvain community detection algorithm (Newman and Girvan, 2004; Blondel et al., 2008; Rubinov and Sporns, 2011) optimally partitioned the graph into communities or clusters of nodes, in each time window, revealing insight into interconnectivity among brain regions and dynamical properties of these connectivity. The communities in the temporal peaks identified by the coarse scale metric are shown in Figure 5*C*, and the brain regions most connected in each local decision were identified.

Fine scale granular information about the graph were obtained at the node level. The in-degrees and out-degrees revealed the sink and source strength of the nodes, in each window.

### Correlations between ${\text{h}}_{\text{γ}}$ power and network features of nodes

To understand the network correlates of the power, we investigated relationships between the network measures: coreness values, in-degrees, out-degrees, and the local power responses. Across multiple nodes, all three network features were found to be correlated with the h*_γ_* power. Brain regions with positive, and negative significant correlations were found, example nodes are shown in Figure 6*A*,*B*, while Extended Data Figure 6-3 shows the evolution of the in-degrees and out-degrees to obtain an intuitive understanding of these correlations. Many negative correlations, especially those in the frontal brain regions, were dominated by sharp decreases in h*_γ_* power in the SA windows that coincided with an increase in the node’s coreness value.

Figure 7 illustrates the locations of the statistically significant Spearman’s correlations between the three network measures and the h*_γ_* power, with 49% of nodes showing correlations with coreness, 43.3% with in-degrees and 34.9% with out-degrees. Most electrodes showed correlations in the “same direction” for all three network features, except for a total of 5 electrodes out of 711 total electrodes for all patients. This analysis was also repeated considering just the SA, and AA windows separately, as different processes govern these two time windows (Extended Data Fig. 7-1). As expected, some brain regions do show opposite directions of correlations in these windows, yet, the overall trend of maximum number of correlations with coreness feature space remains the same.

Finally, we examined the correlation of network features with other narrowband frequency power spectrums, as it is well known that powers in different bands are themselves correlated. The results of correlation of the network features with powers in other bands, particularly the θ (4–8 Hz), α (8–13 Hz), β (13–30 Hz), and γ (30–60 Hz) bands in addition to the h*_γ_* band (h*_γ_*: 60–150 Hz; details in Extended Data Figs. 6-1, 6-2), reveal that it is indeed the case that powers in different frequency bands are themselves related. The electrodes that showed strong positive correlations with h*_γ_* power, also showed strong negative correlations with θ, α, and β power. The significantly correlated electrodes were mostly in pars opercularis and triangularis regions of the left inferior frontal gyrus, also called Broca’s region; as well as motor cortex and superior temporal gyrus (STG) regions.

The results of the correlation analysis indicate that the centrality of a node given by its coreness value is related to the power responses of the node. The results indicate that both increases and decreases in power seem related to how central the node is in the network.

### DCS data

**Figure 8.**
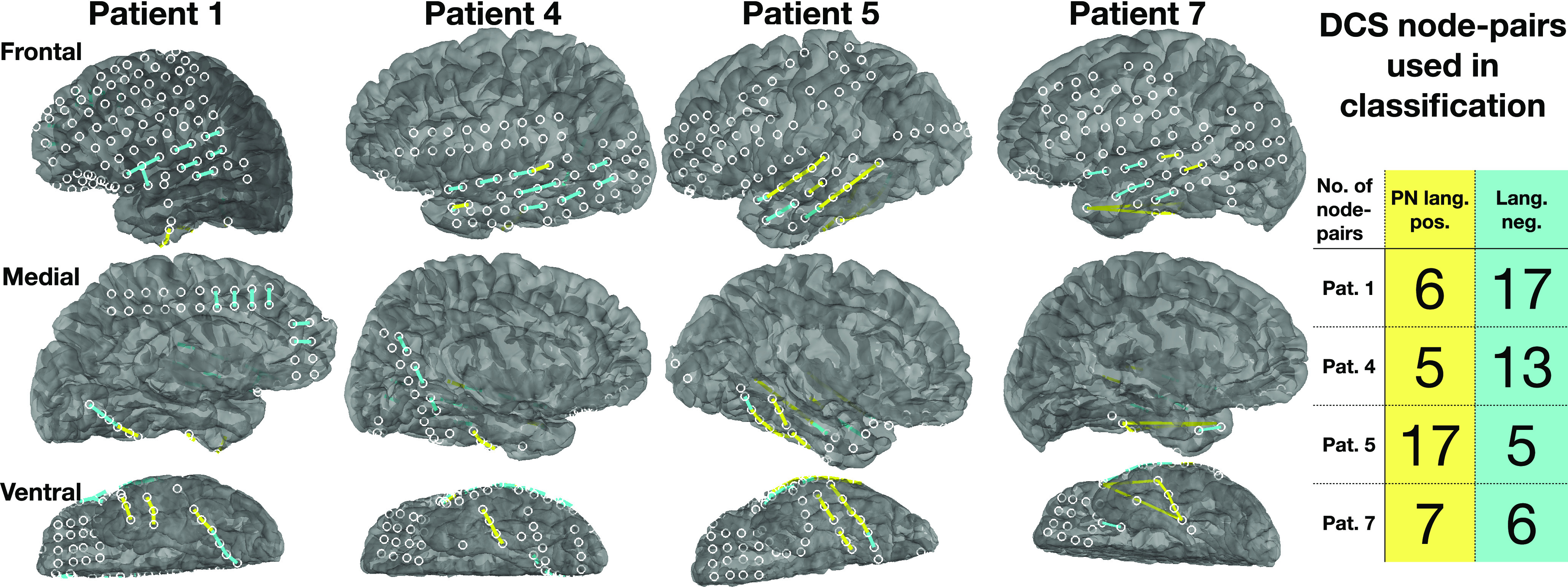
Location of PN positive and language negative node-pairs obtained after DCS. These node-pairs are used as ground truth labels for classification.

DCS is the current gold standard in mapping brain function onto the cortex, before brain resection surgeries. DCS informs the neurosurgeon of language critical areas, to estimate the risk, and potential outcome of the brain resection surgeries. DCS was performed on the same patients as those in whom ECoG data were analyzed, to map out language-specific brain regions before surgery. The DCS data identified which node-pairs were PN positive or language negative, after excluding nodes that had epileptic activity (Fig. 8). DCS data from all seven patients were considered, but only four patients had sufficient DCS node-pairs for classification analysis. Details of all node-pair labels for all patients is given in Table 2.

**Table 2:** Node-pair labels obtained from DCS for all patients

Number of	Total DCS	PN positive	Language negative	Other	Excluded
node-	node-	node-	node-	language positive	node-
pairs	pairs	pairs	pairs	pairs	pairs
**Patient 1**	46	6	17	8	15
Patient 2	25	0	24	1	0
Patient 3	18	1	4	9	4
**Patient 4**	37	5	13	3	16
**Patient 5**	35	17	5	7	6
Patient 6	20	1	3	15	1
**Patient 7**	21	7	6	4	4

The bolded patients are the ones used in the subsequent DCS analysis, as they had sufficient number of node-pairs for analysis.

### Classification results using DCS data as the ground truth

**Figure 9.**
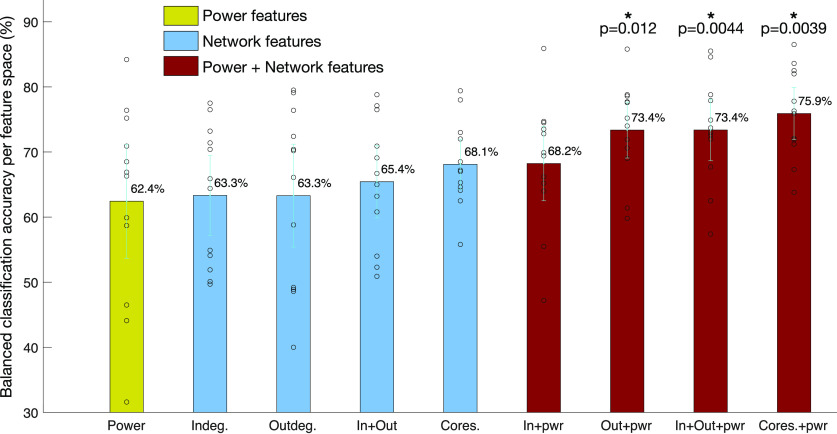
Comparison of binary classification accuracy between PN positive and language negative node-pairs using different feature spaces. The average accuracy in each feature space across all classifiers and patients are shown, with their 95% confidence intervals. While classification accuracy is greater when using network features alone than using the power, it is not significantly greater. However, combining network features and power, “out-degrees+power,” “both in-out degrees+power,” and “coreness+power” perform significantly better than power alone, with “coreness+power” being the best feature space. * indicates *p* < 0.05, the cyan lines indicate the 95% confidence interval, while the black dots are the original data points.

**Figure 10.**
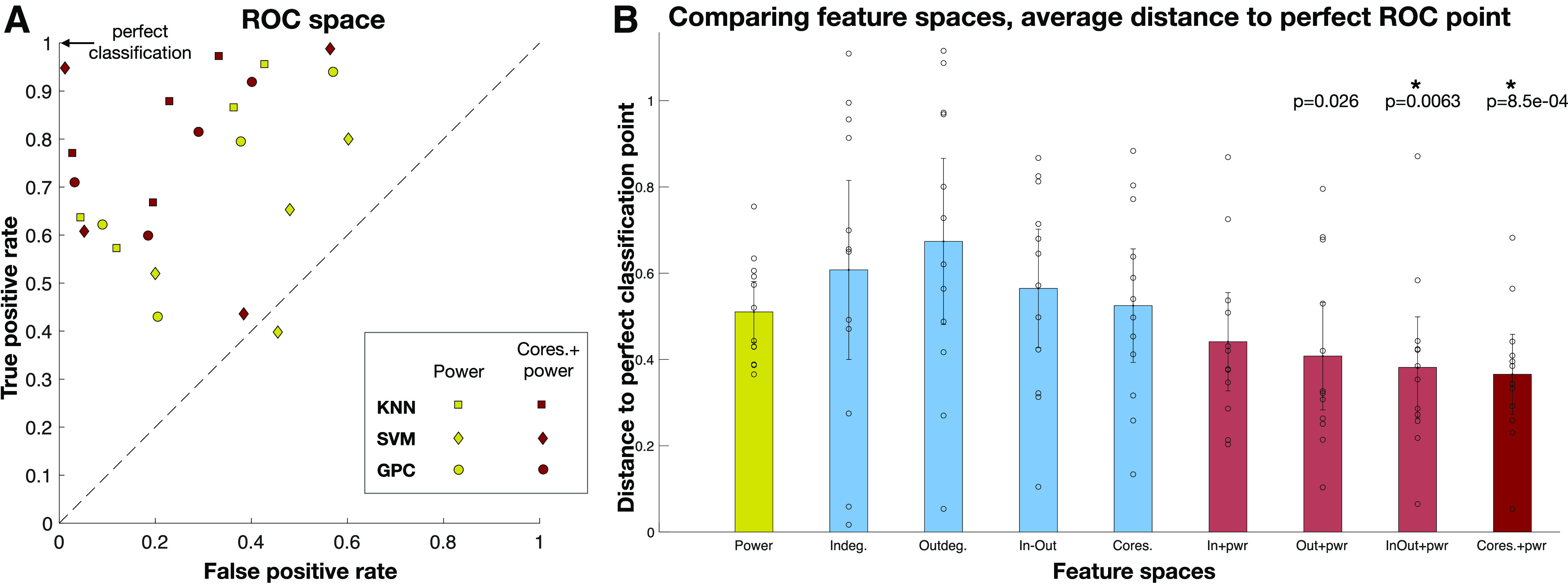
***A***, Each classifier was plotted in the ROC space. TPR = 1, FPR = 0 is the perfect classification point in this space. There are four points for each colored shape, for four patients. ***B***, The average *L*_2_ distance to the perfect classification point, for each feature space is shown in the bar plot, with the 95% confidence interval. Coreness+power feature space is the closest to perfect classification, across all patients, and accounting for classifier variability. Feature spaces “in-out degrees+power” and “coreness+power” perform significantly better than power alone ($p=0.0063,8.5\times 10^{-4}$, *t* test, FDR corrected).

A comparison of classification accuracy across the nine feature spaces was done to reveal insight into which feature space had greater classification accuracy between the PN positive and language negative node-pairs. Three classifiers were used: kNN classifier (Bentley, 1975), linear support vector machine (SVM) classifier (Fan et al., 2008), and the Gaussian process classifier (GPC; Rasmussen, 2003; Rasmussen and Nickisch, 2010) for each of the nine feature spaces, using Python’s Sci-kit learn machine learning toolbox (Pedregosa et al., 2011), to classify between PN positive and language negative node-pairs. Five-fold cross validation was performed with stratified training and test splits, to ensure that classes were balanced. Furthermore, to increase the number of splits while keeping a fixed ratio between training and test set size, repeated random splits of the data were performed 100 times (Varoquaux et al., 2017). The balanced accuracy results of the three classifiers across all test sets are shown in Table 3, with their 95% confidence intervals. Figure 9 summarizes the results from the table, which shows the balanced classification accuracy for each feature space. While all four network features show greater accuracy than power, it is not statistically significantly greater than power. Combined power and network features had higher accuracy; with the highest accuracy feature space being “coreness and power,” statistically significantly greater accuracy than power (*p* = 0.0039, *t* test, significant after FDR correction). To combine results across patients and classifiers, every classifier result was plotted in the ROC space, which plots the TPR versus the FPR. The average distance of classification based on each feature space for all patients and classifiers, to the perfect classification point (TPR = 1, FPR = 0) revealed which feature space had the best classification in the ROC space, shown in Figure 10. The results show that across patients, the feature space coreness+power was closest to perfect classification, significantly closer than the power feature space ($p=8.5\times 10^{-4}$, *t* test, FDR corrected).

**Table 3:** Balanced accuracy results (%) for binary classification between PN positive and language negative node-pairs

Features used for	Patient 1			Patient 4			Patient 5			Patient 7			
KNN, SVM, GPC	*n* = 46			*n* = 36			*n* = 44			*n* = 26			
classifiers (p: length of feature)	KNN	SVM	GPC	KNN	SVM	GPC	KNN	SVM	GPC	KNN	SVM	GPC	Mean
Power……………………….*p* = 104	75.2 ± 1.4	58.7 ± 1.4	68.5 ± 1.3	44.1 ± 0.6	31.6 ± 1.0	46.5 ± 0.7	76.4 ± 1.2	59.9 ± 1.3	70.9 ± 1.4	66.9 ± 1.7	66.3 ± 1.8	**84.2 ± 1.4**	62.4
Indeg………………………..*p* = 104	65.9 ± 1.3	76.5 ± 0.8	73.2 ± 1.5	50.1 ± 0.2	54.9 ± 1.4	51.9 ± 0.7	54.1 ± 1.7	64.4 ± 1.1	49.7 ± 0.2	71.4 ± 1.6	77.5 ± 1.2	70.4 ± 1.7	63.3
Outdeg……………………..*p* = 104	58.8 ± 1.4	79.1 ± 0.7	66.1 ± 1.7	**79.5 ± 1.1**	**70.4 ± 0.7**	49.2 ± 0.4	40.0 ± 1.0	48.6 ± 1.7	48.9 ± 0.7	72.4 ± 1.3	76.4 ± 1.3	70.2 ± 1.5	63.3
Indeg+Outdeg……………*p* = 208	60.8 ± 1.6	78.8 ± 0.7	63.2 ± 1.7	50.9 ± 0.8	69.1 ± 0.8	52.3 ± 0.8	54.0 ± 1.7	66.7 ± 1.5	65.0 ± 1.6	70.9 ± 1.3	77.1 ± 1.2	76.5 ± 1.6	65.4
Cores………………………..*p* = 104	67.2 ± 1.5	**79.4 ± 0.7**	55.8 ± 1.2	64.1 ± 1.5	65.3 ± 1.3	72.0 ± 1.7	62.5 ± 1.5	64.6 ± 1.4	68.5 ± 1.7	**78.0 ± 1.4**	67.0 ± 1.9	73.0 ± 1.7	68.1
Indeg + Power……………*p* = 208	**85.9 ± 1.2**	74.7 ± 1.3	72.8 ± 1.4	69.4 ± 1.5	47.2 ± 1.6	65.2 ± 1.6	73.5 ± 1.5	55.5 ± 1.3	64.0 ± 1.6	66.2 ± 1.4	74.5 ± 1.3	69.9 ± 1.7	68.2
Outdeg + Power…………*p* = 208	78.6 ± 1.2	75.2 ± 1.3	61.4 ± 1.4	74.0 ± 1.4	59.8 ± 1.4	71.9 ± 1.7	78.8 ± 1.4	70.6 ± 1.2	**85.8 ± 1.0**	69.0 ± 1.6	**77.7 ± 1.3**	77.6 ± 1.4	73.4
In+Outdeg + Power……*p* = 312	84.6 ± 1.2	78.8 ± 0.7	72.0 ± 1.5	62.5 ± 1.4	57.4 ± 1.4	73.7 ± 1.5	73.0 ± 1.5	67.7 ± 1.2	85.5 ± 0.9	72.6 ± 1.5	74.9 ± 1.2	77.9 ± 1.3	73.4
Cores. + Power………….*p* = 208	82.5 ± 1.3	77.8 ± 1.1	**75.9 ± 1.3**	63.8 ± 1.6	67.3 ± 1.6	**86.5 ± 1.3**	**82.0 ± 1.0**	**71.2 ± 0.9**	76.3 ± 1.4	71.9 ± 1.5	72.1 ± 1.5	83.6 ± 1.2	75.9

Sensitivity, specificity and precision for each classifier are in Extended Data Tables 3-1, 3-2, 3-3. The bolded entries indicate the feature space which had the highest classification accuracy, for each classifier.

10.1523/ENEURO.0177-20.2020.tab3-1 Extended Data Table 3-1 Sensitivity (recall or TPR), specificity (selectivity or TNR), precision for kNN classification Download Table 3-1, DOC file.

10.1523/ENEURO.0177-20.2020.tab3-2 Extended Data Table 3-2 Sensitivity (recall or TPR), specificity (selectivity or TNR), precision for SVM classification Download Table 3-2, DOC file.

10.1523/ENEURO.0177-20.2020.tab3-3 Extended Data Table 3-3 Sensitivity (recall or TPR), specificity (selectivity or TNR), precision for Gaussian process classification Download Table 3-3, ZIP file.

Finally, we report the sensitivity (also called recall or TPR), specificity (selectivity or TNR), and precision along with their 95% confidence interval for each classifier in the Extended Data Tables 3-1, 3-2, 3-3. The results clearly show greater classification accuracy from a combined power and network feature space, with the best performing feature space being coreness values+power.

## Discussion

Of all the millions of species inhabiting our planet, we *Homo sapiens* are uniquely gifted in our expressive power through language. We effortlessly articulate two to three words per second in fluent speech, yet this deceptively simple task is a highly complex multistage process in our brains (Indefrey and Levelt, 2004; Hickok and Poeppel, 2007). Unfortunately, when disease and brain damage affect such an intricate speech language system, it causes a variety of disorders in millions of people, many of which remain irremediable. Decades of research have greatly enhanced our understanding of these language processes (Dell et al., 1999; Indefrey and Levelt, 2004; Riès et al., 2013; Munding et al., 2016), yet, there exist gaps in our knowledge, particularly in understanding the underlying neural dynamics (Medaglia et al., 2015; Herbet and Duffau, 2020).

Historically, language has been analyzed in a localized manner with a goal of associating cognitive processes to specific brain regions, evidenced by regions’ activation profiles. Recently, language has begun to be studied as a network phenomenon (Bassett and Bullmore, 2009; Fedorenko and Thompson-Schill, 2014; Braun et al., 2015; Forseth et al., 2018; Saravani et al., 2019; Skeide and Friederici, 2016), as it has been theorized that network properties provide greater understanding of the neurobiology of language (Medaglia et al., 2015; Chai et al., 2016).

In prior work, cognitive flexibility is hypothesized as dynamic integration between brain areas in Braun et al. (2015), while Bassett et al. (2015) address the hypothesis that sets of brain regions preferentially interact during a task, and if such interactions differ with learning. Other studies speculate that syntactic processing is distributed across a large ensemble of brain regions (Blank et al., 2016; Xiong and Newman, 2021). All these studies are based on fMRI data. There is an increased focus on studies based on multiscale (Betzel and Bassett, 2017; Domenico, 2017) and modular brain functions (Betzel et al., 2017; Martinet et al., 2020). A review of contributions of network science to cognitive neuroscience using neuroimaging data are in Medaglia et al. (2015), while Herbet and Duffau (2020) is a comprehensive review on the concept of network theory of brain functions. Our work quantitatively evaluated network dynamics using both recording and disruption evidence from ECoG and DCS data.

We interpret “network phenomenon” as multiple physical brain regions, that functionally connect together to subserve a cognitive brain function; and they reconfigure connections as processing goes forward, similar to Salehi et al. (2020). To illustrate with an example; for decades, Broca’s area (inferior frontal regions) was thought to be primarily responsible for speech articulatory processes. While Broca’s area has now been shown to be involved in other cognitive processes as well (Hagoort, 2014; Fedorenko and Blank, 2020); our network view assumes that Broca’s area in conjunction with other brain regions forms a network, and the brain network is responsible for speech articulation.

In this study, we quantitatively measured network dynamics in PN, by assessing the hypotheses: Are different cognitive functions being supported by different network states, or by single regions? In other words, are network measures of brain regions or power profiles of a single region are more predictive of identifying critical language areas?

A majority of previous works using ECoG data have shown that h*_γ_* power is a great indicator of local task activity (Salmelin et al., 1994; Crone et al., 2001; Edwards et al., 2005; Towle et al., 2008; Cogan et al., 2014; Conner et al., 2014; Flinker et al., 2015; Riès et al., 2017). Our data analysis from the PN task from seven patients validated the same phenomenon. Similar to previous work (Kadipasaoglu et al., 2014; Flinker et al., 2015), for each trial, we found strong increases in h*_γ_* power in the visual cortex, aligned to the stimulus onset, and a strong increase in h*_γ_* aligned to the start of articulation time in the pre-motor cortex regions. Furthermore, a strong increase in h*_γ_* in visual cortex in SA windows was accompanied by strong decreases in h*_γ_* power in some frontal regions, for all patients. Such patterns of power activations and deactivations were seen in many brain regions during the task. The NetDI methodology allowed us to obtain network dynamics among these brain regions, and we asked the following questions: (1) Is there a relation between the network measures and the local power responses? (2) Do the network measures contain any additional information about the language processes, compared with power? The answers to these questions are indicative of the original hypothesis, about whether cognitive functions are supported by brain networks, or local brain regions. To answer the first question, we investigated relationships between h*_γ_* and the network measures, to understand the various aspects of the relationships. To answer the second question, we performed a classification analysis between PN positive and language negative node-pairs, considered as ground truth, using DCS data (Ojemann et al., 1989; Sinai et al., 2005), for the same set of patients for whom the NetDI analysis was done.

In order to understand the network basis of power responses, we developed a graph theoretic framework NetDI, to discern the spatiotemporal brain dynamics underlying language. DI provided a robust information theoretic measure of causality, even if the underlying data were nonlinear. A multiscale graph theoretic analysis revealed network properties of the process at various scales. The coarse scale analysis revealed task related peaks in temporal dynamics conjectured to be local processing “stages” in the brain, because of their strong alignment with task activities. These stages could correspond with separable cognitive processes that are thought to be invoked during speech production (Levelt, 1989; Salmelin et al., 1994). At an intermediate scale, k-cores and the Louvain analysis provided alternate spatiotemporal views of the process. K-cores uncovered the highly interconnected innermost core of the network, providing “coreness values” for each node, as a measure of centrality of the node in the network. The Louvain analysis revealed a distributed overlapping set of communities with very strong interconnectivity within each community, consistent with recent suggestions toward interactive networks in Betzel and Bassett (2017), Saravani et al. (2019), and Martinet et al. (2020). The fine scale analysis revealed the out-degrees (source strength) and in-degrees (sink strength) of the nodes, time-varying network measures that provide additional insight into the language process.

Armed with these network measures, we sought to understand whether there existed a relation between the network measures and the local power responses. All three network features, coreness values, in-degrees and out-degrees were significantly correlated with the h*_γ_* power, with most electrodes showing positive and negative correlations with coreness values. The network measure coreness of nodes values is a measure of the centrality of the node, and typically depends on both in-degrees and out-degrees. Our results align with prior work that indicated coreness of nodes as a good measure of a node’s influence in the network (Kitsak et al., 2010; Pei et al., 2014; Shin et al., 2016). In Xiong and Newman (2021), language processing was found to be supported by activation of both core networks, and periphery brain regions. Our results show that a region’s coreness and power features contain different information about the language process. This is substantiated by the fact that there were both positive and negative correlations, which could indicate different processes where the centrality of the node was related to either the increase or decrease in h*_γ_* power. Furthermore, differences in correlations between SA and AA windows also existed, along with different fine-scale measures that drove the correlations. It seems that the information content of in-degrees, out-degrees and coreness values are related to h*_γ_* power, but not equivalent.

In order to quantitatively verify whether the network measures contain additional information from power, the data from DCS were used to classify language areas as critical to processing (PN positive) or not (language negative) using feature spaces consisting of power, network measures and a combination of power and network feature spaces. The classification results across patients and multiple classifiers indicate superior classification of combined network and power features compared with power alone. The feature space consisting of coreness of nodes and power emerged as the best classifier, thus indicating that the centrality of the node in the network is an important feature for understanding language dynamics. This confirms the hypothesis that network features do contain provide novel information about the language process beyond information given by power. Our results agree with other studies like (Jiang et al., 2020; Salehi et al., 2020) where functional networks dynamically are hypothesized to reconfigure based on cognitive states, in an individualized manner. Extensive recent work in aphasia studies suggest that patients with seemingly different lesion locations could experience similar impairments, probably because the lesions affect a broad cortical network needed for the language task (Fridriksson et al., 2018). Our results provide further evidence that the centrality of the brain region has a critical role to play in the language system, beyond the local processing of the region.

### Future research directions

(1) The h*_γ_* power in ECoG data are a component of the local field potential signal, which is inherently ambiguous because of contributions from multiple sources like synaptic inputs, spikes and volume conduction, making them harder to interpret. Further studies could elaborate on the various subcomponents of the network basis of power. Furthermore, it would be interesting to study the differences between the various frequency bands, similar to Lam et al. (2016), in terms of discriminability between language critical and language negative areas, to understand spectral components of the language process.

(2) The examination of Louvain communities indicates that the PN positive brain regions had strong connectivity with distant brain regions. Within the Louvain communities, the was a lack of connections between the node-pairs themselves, the nodes involved in PN positive areas were instead connected with distant brain regions. We speculate that DCS did not just disrupt a local process, but rather disconnected the local brain region from downstream brain regions, however further studies need to be done to prove or disprove that.

(3) In future work, NetDI could be used to improve pre-surgical DCS language mapping in patients (Szelényi et al., 2007). DCS has some unpleasant side-effects, it sometimes induces seizures and has after-discharge effects, so the doctors would prefer to test only as many regions as is essential for clinical mapping. Network and power measures can be found for all nodes, from experiments before performing DCS. Given a few ground truth node-pairs, the corresponding network measures could be used as training data to identify other potential language positive and negative areas, to guide doctors in making DCS more clinically efficient.

(4) This work is important in understanding why certain focal brain lesions cause widespread disruption of the networks of the brain (Gratton et al., 2012; Fridriksson et al., 2018). Overall, NetDI has the potential to relate brain cognitive theories of language with the neural connectivity patterns, and can validate cognitive theories of language (Dell, 1988; Dell et al., 1999; Bassett and Bullmore, 2009; Salehi et al., 2020).

10.1523/ENEURO.0177-20.2020.f6-1 Extended Data Figure 6-1 A matrix representation of correlation coefficients of in and out degrees with powers in five frequency bands are shown for each of the seven patients. Rows of all matrices are electrodes grouped by brain region, and there are 10 columns for each patient. First two columns are correlation coefficient of in-degrees and out-degrees with h*_γ_* power (60–200 Hz), next two columns are in/out-degrees with γ power (30–60 Hz), next two columns are correlation coefficient of in/out-degrees β power (13–30 Hz), next two columns are correlation coefficient of in/out-degrees α power (8–13 Hz), while the last two columns are with θ power (4–8 Hz). It can be observed that while the electrode coverage of all the patients is different, there exist similar trends of high positive correlation with *h_γ_* power and negative correlation with the θ power, particularly in language-related brain regions. The brain regions are labeled with numbers and are enumerated in Extended Data Figure 6-2. The exact number of electrodes that were positively and negatively correlated for in-degrees and out-degrees are also shown, and more electrodes were found to be negatively correlated with out-degrees than in-degrees, while greater number of electrodes are positively correlated with in-degrees than out-degrees. Download Figure 6-1, EPS file.

10.1523/ENEURO.0177-20.2020.f6-2 Extended Data Figure 6-2 Table depicting the brain regions and number of recording electrodes in each region for all patients. Download Figure 6-2, TEX file.

10.1523/ENEURO.0177-20.2020.f6-3 Extended Data Figure 6-3 ***A***, A positively correlated occipital-temporal-lateral-fusiform-gyrus electrode from patient 5. ***B***, A negatively correlated Inf-temporal electrode from patient 5. The colors of the nodes in ***A***, ***B*** represent the power in the window, while the dark blue lines are in-degrees to the node, and the light blue lines are the out-degrees. These figures demonstrate that coreness of nodes is due to the combined effect of in and out degrees and provide a pictorial understanding of how in/out degrees correlates with power. Download Figure 6-3, EPS file.

10.1523/ENEURO.0177-20.2020.f7-1 Extended Data Figure 7-1 Every three-ring electrode on the brain denotes the correlation coefficient value of three feature spaces with power. The significant correlation coefficient of in-degrees and h*_γ_* power time-series are shown in the innermost circle, the correlation coefficient of out-degrees with power is denoted by the color of the middle ring, while the outer ring for each electrode’s color denotes the correlation coefficient of coreness of nodes with power. The absence of color in the outer ring, or the absence of either the middle or inner ring denotes the lack of significant correlation in that electrode, with that feature space. The first row denotes the correlation coefficient calculated using the entire time-series, and is the same as the main figure in the paper. The second row shows the correlation coefficient of the three feature spaces with power, when only the SA time windows are considered, while the third row shows the correlation coefficient when only the AA windows are considered. The bar plots to the right denote the average percentage of electrodes that showed significant correlation for each feature space, after correcting for multiple comparisons (FDR, *p* < 0.05 for each feature space, per patient). It can be noted that most electrodes’ feature spaces show correlation in the same direction. There are electrodes where the correlation in SA and AA are in the opposite directions, and are canceled out in the combined SA+AA correlation space. Overall, more electrodes have significant correlation of coreness of nodes feature space with power, than the other two feature spaces, in all three various time-window considerations. Download Figure 7-1, EPS file.

## Synthesis

Reviewing Editor: Satu Palva, University of Helsinki Decisions are customarily a result of the Reviewing Editor and the peer reviewers coming together and discussing their recommendations until a consensus is reached. When revisions are invited, a fact-based synthesis statement explaining their decision and outlining what is needed to prepare a revision will be listed below. The following reviewer(s) agreed to reveal their identity: NONE.

In the paper “NetDI: Non-linear cortical dynamics underpin word production” the authors investigate the cortical basis of a picture naming (PN) task. They aim at understanding whether the computations underlying the PN task are well-localized withing brain regions or rather distributed among regions. To answer this question they use data from intracranial electrocorticography (EcoG) in the high gamma range as well as direct cortical stimulation (DCS). They use information and graph theoretic approaches to analyze the ECoG data and develop a framework they named: “Network dynamics using Directed Information (NetDI)", which allowed them to study the properties of graphs constructed based on directed information between nodes at different times during the task. The authors find that an information-graph theoretic measure, the node in-degree time course, is correlated (sometimes positively, sometimes negatively) to the high-gamma power time course in a large fraction of nodes.

They also found that information-graph theoretic measures (in particular coreness of nodes) together with the high-gamma power better differentiate between language positive vs negative DCS node pairs than high-gamma power alone. They conclude that the network features provide information about the necessity of different brain regions for this PN task that is not contained in the high-gamma power alone.

This is a technically sound manuscript that investigates an important question regarding how network dynamics contribute to the necessary involvement of a cortical region in processing language. However, the “big picture” on how the different analyses were motivated, how they fit together, and what conclusions can be derived from them is missing. Further, the writing is often convoluted or confusing, and should be more concise and emphasize the important aspects. There were also a few issues with regarding the language that should be corrected. The authors should improve the overall structure and message of the manuscript, and perhaps also reduce the number of main figures.

Major

The “ big picture” in the manuscript is unclear, i.e. how the different findings at different scales fit together, what likely conclusions are there, what possible conclusions, and what should be further investigated in future research?

Why was only gamma-band power investigated, and why are oscillations in other frequency bands not even mentioned in the manuscript?

While employing both information and graph theoretic measures together with power estimates to predict critical regions is a valid approach, the conclusion that “such network parameters are more predictive than gamma power for identifying critical language regions in the brain” is not accurate. Based on Figures 10 and 11, network features can actually provide worst predictions compared with power features which pr comparably (Pt 4 Figure 10) or even perform better, for example distance to perfect ROC is lower for power in figure 11. However, the reviewers agree that the combined information of network and power estimates provide superior performance (in most cases). The power alone and network features alone should also be shown here. The fact that adding more features gave a worse result (cores+power vs cores+edge+power) might indicate that there is a problem with overfitting.

While the network dynamics are intriguing and can provide insights, the strength of the paper is in elucidating the features that differ from high gamma power and how these can be leveraged and related to DCS mapping. I urge the authors to expand their analyses on how (and why) network features can either correlate with high gamma both positively and negatively and how that is mapped out on cortex.

The predictions accuracies for DCS seem noisy in the sense that the average accuracy (table 3, figure 10) are many times very similar across feature sets. Taken together with the fact that a leave one out validation approach has a large confidence interval (Varoquaux et al 2017) it is hard to assess significant differences across feature sets (and models). I would recommend adding the variance across folds and using a lower CI biased validation such as 5 (or more) CV. It would strengthen the manuscript to add more DCS patients if available (if not then the limitation of a low N should be discussed) and to focus on how network features plus power estimates provide a better mapping (instead of stating network features are superior) and then to discuss the interpretation.

The disconnection theory in line 308-309 (Figure 12) is intriguing but is purely speculative and there is no statistics or data backing it. This should be removed or addressed in the discussion rather as a main result unless further data analysis can be provided.

The discussion of prior findings, especially with regards to networks, seems a bit short, while the discussion of DI seems long and could be made more concise. The motivation underlying the analyses performed here should be made clearer.

p.4: the Methods section should start with a description of the task, which seems to be missing completely.

p.13, l. 197-206: This section is unclear. Were several correction methods for multiple comparisons used? Please rewrite for clarity.

p.17, l. 253-59: This section is confusing. Positively and negatively correlated electrodes should be distinguished more clearly and numbers about the prevalence of the latter given, as well as statistics for correlations of out-degree with gamma power. I am not sure if the figure legend in Figure 6 actually represents the findings.

Would it be possible add some measure of uncertainty for Tables 3 and 4?

Figure 4 lacks clarity. It might be informative to show the actual matrices that were used as adjacency matrices for calculating all the graph theoretic measures, i.e., after thresholding the “change in DI from baseline” matrices. It would also be good to add a comment about the all the negative values that are being discarded, that might be informative?

How does the max pooling in each category affect significance testing? Was there a significance testing done to claim that network features alone is better than power features alone (i.e., yellow vs blue)?

Is any significance testing possible for Table 4?

It is hard to understand how significant the result of Figure 12 is. What would expected for a random node. Also how do the AA windows behave? C legend is also missing.

Minor

The title is not very informative about the study. NetDI is a method, which doesn’t make it clear. In addition although the authors use information theory, which picks up linear and non-linear relationships between nodes, there is no in-depth discussion about non-linearity in the paper - and the authors do not directly show that a purely linear method would not perform as well, which makes the claim that “non-linear cortical dynamics underpin word production” (as opposed to linear?) not very substantiated.

p.4, l.69: please explain briefly by which criteria how were “significantly noisy” channel identified?

p.5: that 0 ms can refer to either stimulus onset or articulation time should be explained more clearly in the text, and not in a footnote

p.8, l.103: it should be explained at the beginning of this section and more clearly what causes bias, and why the used methods correct for it

p.8. l.103: from here on, the line numbering is faulty

p.9: the section about thresholding should be rewritten for clarity

p.11 the section about Louvain clustering seems excessively verbose and could be condensed

p.12, l.173: do you mean “any of the 3 language tasks”?

p.14, Figure 4: Please rename the “from” and “to” electrodes, e.g. as “source” and “target” or similar.

p.16, Figure 5: While the general idea of this figure is good, having “-256 to 0” below the x-axis in A is confusing

p.17, l.263: What is the relationship here between DCS and brain resection surgeries?

p.23, l. 322-332: are these two possible interpretations necessarily mutually exclusive?

p.23, l. 332-337: these should be in the same paragraph, and in condensed form.

p.24, l.386-393: It is not clear how NetDI would influence DCS in practice.

The results of Figure 6 are not discussed. How does out-degree behave in relation to high gamma,

and how to interpret the negative correlation of high-gamma and in-degree?

Line 286: “(1) For 3 out of 4 patients, the Gaussian Process Classifier had the highest accuracy, and was thus identified as the best classifier.” Could the authors substantiate this claim, maybe by adding an additional row in Table 3, showing the mean accuracy for each patient and classifier.

Line 304: Is it the correct that after the Louvain clustering many nodes were not inside any community? i.e., community of size 1, as at initialization? It might then be helpful to show on figure 12A the percentage of nodes that are within a community, to understand how significant the finding is that a lot PN-positive nodes are within a community.

Line: 306: Are the authors are claiming that the node-piars that are PN positive, are usually nodes that are part of 2 different Louvain communities? Can this be quantified?

## Author Response

Revision statement

--------------------------------------------

Manuscript Instructions

- The species studied is not mentioned in the abstract. Please make sure to update both the abstract in the article file and on the submission form.

Authors: We thank the reviewers for pointing this out, we have now updated the abstract to clarify that the intracranial data is collected from humans.

Synthesis Statement for Author (Required):

In the paper “NetDI: Non-linear cortical dynamics underpin word production” the authors investigate the cortical basis of a picture naming (PN) task. They aim at understanding whether the computations underlying the PN task are well-localized withing brain regions or rather distributed among regions. To answer this question they use data from intracranial electrocorticography (EcoG) in the high gamma range as well as direct cortical stimulation (DCS). They use information and graph theoretic approaches to analyze the ECoG data and develop a framework they named: “Network dynamics using Directed Information (NetDI)", which allowed them to study the properties of graphs constructed based on directed information between nodes at different times during the task. The authors find that an information-graph theoretic measure, the node in-degree time course, is correlated (sometimes positively, sometimes negatively) to the high-gamma power time course in a large fraction of nodes.

They also found that information-graph theoretic measures (in particular coreness of nodes) together with the high-gamma power better differentiate between language positive vs negative DCS node pairs than high-gamma power alone. They conclude that the network features provide information about the necessity of different brain regions for this PN task that is not contained in the high-gamma power alone.

This is a technically sound manuscript that investigates an important question regarding how network dynamics contribute to the necessary involvement of a cortical region in processing language. However, the “big picture” on how the different analyses were motivated, how they fit together, and what conclusions can be derived from them is missing. Further, the writing is often convoluted or confusing, and should be more concise and emphasize the important aspects. There were also a few issues with regarding the language that should be corrected. The authors should improve the overall structure and message of the manuscript, and perhaps also reduce the number of main figures.

Authors: We are grateful to the reviewers for their detailed comments and feedback. We have addressed all the comments, we have detailed responses to each comment below.

----------------------------------------

Major

----------------------------------------

1) The “ big picture” in the manuscript is unclear, i.e. how the different findings at different scales fit together, what likely conclusions are there, what possible conclusions, and what should be further investigated in future research?

Authors: We thank the reviewers for commenting on the missing big picture, and how the various findings tie in together. We did more analysis, both in understanding correlations with various feature spaces, and classification with DCS using more feature spaces. We now clearly demonstrate the various types of correlations that exist with 3 network features - coreness of nodes, in-degrees and out-degrees. Coreness of nodes is an aggregate measure that combines information from in-degrees and out-degrees, and is indicative of the node’s influence in the network. Coreness values could be dominated by in-degrees or out-degrees, both fine scale measures, based on the brain region. We also expanded the classification of language critical regions based on DCS data, by including more feature spaces, and clearly demonstrates that network features combined with power provide greater discriminability among the language critical areas. We tie in our results , and provide the motivation for the various analysis in the discussion section. We also include a section where we discuss “future research directions", and discuss possible findings that merit further investigation.

2) Why was only gamma-band power investigated, and why are oscillations in other frequency bands not even mentioned in the manuscript?

Authors: We thank the reviewers for bringing this up. Indeed, when we had first started our analysis, we explored the effects of other frequency bands and network features. We had restricted our results to high-gamma, due to the vast literature that demonstrates the correlation between the task related phenomenon and high-gamma power. In the new revision of the paper, we include Extended Figure 6-1 and Extended Table 6-1 to show the correlation of in-degrees and out-degrees with four frequency bands :high-gamma 60-200Hz, gamma power 30-60Hz, beta power 13-30Hz and theta power 4-8Hz. We found that a strong positive correlation of a feature space with high-gamma corresponded with a strong negative correlation with theta power. This is an observed phenomenon, a strong increase in high -gamma activity is typically accompanied by a suppression of theta activity. We present this figure and table to describe the phenomenon and highlight the brain regions of the cortex across all 7 patients that have the highest correlation coefficients. In this work, we only show the relationship between network features and high-gamma, which the most commonly used feature space for understanding language in ECoG. We believe there can be potentially future directions that can be explored by further understanding the effects of other frequency bands in the processing of language dynamics, and we discuss that in the future directions.

3) While employing both information and graph theoretic measures together with power estimates to predict critical regions is a valid approach, the conclusion that “such network parameters are more predictive than gamma power for identifying critical language regions in the brain” is not accurate. Based on Figures 10 and 11, network features can actually provide worst predictions compared with power features which pr comparably (Pt 4 Figure 10) or even perform better, for example distance to perfect ROC is lower for power in figure 11. However, the reviewers agree that the combined information of network and power estimates provide superior performance (in most cases). The power alone and network features alone should also be shown here. The fact that adding more features gave a worse result (cores+power vs cores+edge+power) might indicate that there is a problem with overfitting.

Authors: We are very thankful to the reviewers for such a careful review and providing excellent feedback to improve the paper. Based on this and other review comments, we expanded our feature space to include more network features individually (in-degrees, out-degrees, coreness, in-out degrees and edge-betweenness centrality). We found that edge-centrality feature space performed poorly for all patients and classifiers, and combining edge with other feature spaces brought down the performance of the feature space. In other words, coreness + edge. feature space performed worse than coreness alone. Based on this new careful analysis, that included confidence intervals from 5-fold cross validation results, we have discarded edge betweenness centrality as a feature space, as it did not add any value to the classification. We now look at 9 feature spaces: (1) power, (2) in-degrees (3) out-degrees (4) in-deg+ out-deg, (5) coreness of nodes (6) in-degrees + power, (7) out-degrees + power (8) indeg+outdeg + power (9) coreness + power. Thus, we look at 3 categories of feature spaces: power feature space, 4 network feature spaces, and 4 network + power feature spaces. We are now able to show that all 4 network + power feature spaces perform better than power alone, with the most significant one being coreness of nodes + power feature space.

4) While the network dynamics are intriguing and can provide insights, the strength of the paper is in elucidating the features that differ from high gamma power and how these can be leveraged and related to DCS mapping. I urge the authors to expand their analyses on how (and why) network features can either correlate with high gamma both positively and negatively and how that is mapped out on cortex.

Authors: We thank the reviewers for their insightful comments. The reviewers rightly ask, “how and why network features correlate with high gamma", and to answer that, we have done further analysis. In the previous version of our paper, we had shown that in-degrees of electrodes correlated with power (both positively and negatively), for multiple electrodes. We mentioned that out-degrees also correlated, but in fewer numbers, and did not show that in the paper. We now expanded our analysis to include the feature space ``coreness of nodes’, and calculated correlations of coreness of nodes with power. Surprisingly, we found that a greater number of electrodes correlated with coreness of nodes, than either in-degrees or out-degrees. The new Figure 6 now shows the correlation of all three features spaces on the cortex. We can see that the correlation of all 3 features tend to be in the same direction (all three features are positively or negatively correlated with power), except a total of 5 electrodes across all 7 patients, where the corr. coeffs. of indeg and outdeg are in the opposite directions. We then asked the question that since the SA time windows and AA time windows can have very different brain processes, it might be informative to separate the time windows and calculate correlations in SA and AA windows separately. We include the results of this new analysis as an extended Figure 7-1, that shows the correlation for all time windows (also in the main figure), the SA windows and AA windows separately. The key finding is that more electrodes correlate with coreness of nodes than the other two features, for all sets of time windows. Coreness of a node is related to the max core network that node belongs to, and is an integer value. It is related to the total degree of the node, and thus combines information from both in-degrees and out-degrees, in a broader sense. Coreness of nodes is an intermediate scale metric, that has been shown to be related to the total degree of a node. Thus, the power increase/decrease of an electrode seems to be related to how highly connected the node is in the network. We found that for all 7 patients, the magnitude of the highest positive correlated electrodes for in-degrees coincided with Broca’s region (frontal inferior opercularis and triangularis) and temporal regions. We also found that the strongly negatively correlated electrodes are typically due to a sharp decrease in power in the SA time windows, with a simultaneous increase in in/outdegrees. Some examples of positive and negative correlations are shown in Figure 6 in the main paper, and some pictorial representations in Extended Figure 6-2. Further analysis with DCS revealed that these network features have more information about the underlying processes than classical power. (described in response to other comments).

5) The predictions accuracies for DCS seem noisy in the sense that the average accuracy (table 3, figure 10) are many times very similar across feature sets. Taken together with the fact that a leave one out validation approach has a large confidence interval (Varoquaux et al 2017) it is hard to assess significant differences across feature sets (and models). I would recommend adding the variance across folds and using a lower CI biased validation such as 5 (or more) CV. It would strengthen the manuscript to add more DCS patients if available (if not then the limitation of a low N should be discussed) and to focus on how network features plus power estimates provide a better mapping (instead of stating network features are superior) and then to discuss the interpretation.

Authors: The authors thank the reviewers for this excellent suggestion and feedback. We had previously not considered 5-fold CV, due to the limitations of low number of labelled node-pairs (N). The lowest N out of the 4 patients was 13, and the data available for training reduced the accuracy across all feature spaces. Fortunately, we were able to perform data-augmentation, and double our available node-pairs in a robust manner. For example, if we considered the power feature space, a row in the classification matrix consisted of the time-series of power of one node followed by the time series of another node from the node-pair. For each node-pair, we created another labelled node pair by reversing the order of the time-series of the node-pairs. This gave us enough data to perform 5-fold cross validation, and have stratified training and test splits, to have balanced classes for training and testing. We found that the balanced accuracy results from the leave-one out cross validation prior to data augmentation, and from the 5-fold cross validation after data augmentation, had similar trends across feature spaces, for all classifiers except the Decision Tree Classifier (DTC), which performed poorly for all feature spaces after data augmentation, and was thus excluded from the paper. We also excluded the network feature based on “Edge betweenness centrality", since classification was poor for the Edge feature by itself, and combining it with any other feature space made the classification worse, and the feature doesn’t seem relevant. We now analyze network features: in-degrees, out-degrees, combined In-out degrees, and coreness of nodes individually, and the same network features combined with power. The new results clearly demonstrate improved classification accuracy upon combining power and network features, compared to just power alone.

6) The disconnection theory in line 308-309 (Figure 12) is intriguing but is purely speculative and there is no statistics or data backing it. This should be removed or addressed in the discussion rather as a main result unless further data analysis can be provided.

Authors: We agree with the reviewers that we need further analysis to back up the claim, and have removed this figure from the main paper, to stick to the main finding in the paper, in which we prove that addition of network features improves the classification accuracy between language positive and negative regions. We discuss potential future works that can address these theories.

7) The discussion of prior findings, especially with regards to networks, seems a bit short, while the discussion of DI seems long and could be made more concise. The motivation underlying the analyses performed here should be made clearer.

Authors: We thank the reviewers for highlighting this shortcoming in our previous version of the paper. We have now expanded our introduction, and the discussion sections, to make the motivation clearer, and added some prior literature with regards to the networks. We have also made the discussion on DI more concise, to improve clarity.

8) p.4: the Methods section should start with a description of the task, which seems to be missing completely.

Authors: We thank the reviewers for pointing this out, we realize that the caption of Figure 1A was insufficient as a task description. We have now added the task description at the start of the Methods section.

9) p.13, l. 197-206: This section is unclear. Were several correction methods for multiple comparisons used? Please rewrite for clarity.

Authors: We thank the reviewers for the feedback. We have now rewritten this section for clarity. The reported Louvain communities were bon-ferroni corrected. All other multiple comparison performed were FDR corrected.

10) p.17, l. 253-59: This section is confusing. Positively and negatively correlated electrodes should be distinguished more clearly and numbers about the prevalence of the latter given, as well as statistics for correlations of out-degree with gamma power. I am not sure if the figure legend in Figure 6 actually represents the findings.

Authors: We agree with the reviewer’s feedback that the section about correlated electrodes needs improvement. The previous Figure 6 did indeed represent the findings, we only show the electrodes with significant correlation on the cortex, and the table represented the percent of electrodes that had the correlation. However, we realize that it could be made clearer. In order to answer this comment, along with addressing comments (1) and (4), we evaluated relationships between 3 network features and high gamma power - coreness values, in-degrees and out-degrees. We found - correlations existed between all 3 feature spaces and power, mostly in the same direction for all 3 feature spaces per node. Comparing number of electrodes with significant correlation in each feature space, we found coreness values > in-degrees > out-degrees. We created a new Figure 6, with detailed statistics for each feature space, with a bar plot and 95% confidence intervals. Extended Figure 6-1 shows the exact number of positively and negatively correlated electrodes for indegrees and outdegrees, per patient. The biggest take-away is that the negative correlation most often occurred due to a power decrease in the SA windows, accompanied by an increase in the coreness value (which is due to an increase in indegrees or out-degrees, based on the node). A pictorial representation of these correlations is shown in Extended Figure 6-2, to provide more intuition.

11) Would it be possible add some measure of uncertainty for Tables 3 and 4?

Authors: We thanks the reviewers for this excellent suggestion. We now include 95% confidence intervals for these tables. We needed more data to be able to generate confidence intervals, and for that, we performed data augmentation in a principled manner, which allowed us to double the labelled node-pairs for classification. The table 3 now has balanced accuracy results from 3 classifier results KNN, SVM and GPC, for 9 feature spaces, with a 95% confidence interval. The 5-fold CV was repeated on 100 random splits of the data, to obtain the measure of uncertainty. Table 4 previously showed the sensitivity, specificity and precision for just the GPC classifier. It has now been removed from the main paper, and is now replaced by 3 tables in the Extended data, showing the sensitivity, specificity and precision for all 3 classifiers. The tables include a 95% confidence interval for each of the classification parameters.

12) Figure 4 lacks clarity. It might be informative to show the actual matrices that were used as adjacency matrices for calculating all the graph theoretic measures, i.e., after thresholding the “change in DI from baseline” matrices. It would also be good to add a comment about the all the negative values that are being discarded, that might be informative?

Authors: We thank the reviewers for very helpful feedback in improving the clarity of Figure 4. Previously, we had shown the graph-time series using the “change in DI matrices” and indeed it would greatly improve the figure to show the actual thresholded matrices that were used in the analysis. We have now modified Figure 4 to show examples of all three matrices, the original DI matrix, the change in DI matrix that shows both the positive and negative changes, and the final thresholded matrix that retains only the top positive increases in DI. We have also added a comment in the methods section under “Multi-scale graph theoretic framework” to address that we are only retaining the positive values, the final results are to be interpreted within this framework. Indeed, the negative values that are discarded might be informative too, though interpretation of results with a network having both positive and negative directed edges becomes more challenging. Our observations of the DI matrices revealed that a cluster of nearby electrodes, particularly in the orbital frontal cortex typically have less information flow among each other (negative changes in DI) at certain times of the task, and more information flow to other brain regions. Based on such observations and conversations with experts, we considered it a reasonable assumption to look at interpreting task related changes based on “increases in information flow”. Future research could definitely address more complex network edges that consider both positive and negative information flows.

13) How does the max pooling in each category affect significance testing? Was there a significance testing done to claim that network features alone is better than power features alone (i.e., yellow vs blue)?

Authors: We thank the reviewers for pointing out the effect of max-pooling on significance. We had previously shown the accuracy results in each category for just the GPC classifier, by max-pooling for the best feature space in the “network space” category, and the combined “network+power space” category. In the new version of the paper, we no longer perform max-pooling. We now show the bar plot of average accuracy of each feature space, across all classifiers and patients (the new Figure 9). We perform significance testing by using the t-test, comparing the accuracy of each feature space with power. Since we compare 8 feature spaces with power, we correct for multiple comparisons, and we obtained 3 feature spaces in the “power + network” category, that had significantly greater accuracy than power.

14) Is any significance testing possible for Table 4?

Authors: In this new revision of the paper, we have replaced Table 4 with three tables in the Extended data (Table 3-1, 3-2 and 3-3), that show the Sensitivity, specificity and precision for each of the 3 classifiers used in the paper. We now show a 95% confidence intervals for each parameter, obtained from repeated k-fold cross validation. We could test for significance by shuffling class labels. However due to small N limitations, and overall, unequal class sizes, we do not perform significance testing for each parameter. Permutation testing by shuffling labels is reliable only when equal class sizes are used. Since the goal was to compare feature spaces, we instead test for significance when comparing feature spaces’ to the power feature space.

15) It is hard to understand how significant the result of Figure 12 is. What would expected for a random node. Also how do the AA windows behave? C legend is also missing.

Authors: We thank the reviewers for the very valid feedback about this Figure. We were trying to use the knowledge of known DCS nodes, to understand when in the network they were most connected. Based on comment 6, we decided to take the reviewer’s feedback and eliminate figure 12 from the paper, and discuss potential implications about the role of PN positive node -pairs in the network as future extensions of the work.

----------------------------------------

Minor

----------------------------------------

1) The title is not very informative about the study. NetDI is a method, which doesn’t make it clear. In addition although the authors use information theory, which picks up linear and non-linear relationships between nodes, there is no in-depth discussion about non-linearity in the paper - and the authors do not directly show that a purely linear method would not perform as well, which makes the claim that “non-linear cortical dynamics underpin word production” (as opposed to linear?) not very substantiated.

Authors: We completely agree with the reviewers that the current title doesn’t make it clear that NetDI is a method, and DI does indeed capture both linear and non-linear relationships. We have now changed the title to “NetDI: Methodology elucidating the role of power and dynamical brain network features that underpin word production”.

2) p.4, l.69: please explain briefly by which criteria how were “significantly noisy” channel identified?

Authors: We thank the reviewer for bringing this up. The words “Significantly noisy” channels are those which included epileptiform activity. These records were reviewed by an experienced researcher, the attending neurologist in a clinical capacity, and the attending neurosurgeon is listed as an author. We have modified the text in the paper, and replaced “were significantly noisy” with “included epileptiform activity", to make it more clear.

3) p.5: that 0 ms can refer to either stimulus onset or articulation time should be explained more clearly in the text, and not in a footnote

Authors: We have now included this information as part of the main text, and not as a footnote.

4) p.8, l.103: it should be explained at the beginning of this section and more clearly what causes bias, and why the used methods correct for it

Authors: We thank the reviewers for the suggestion to improve the clarity on the cause of estimator bias. Indeed, all estimators have a bias, due to the finite nature of the amount of available data. Ideally, when data sizes tend to get larger, the estimator bias tends to zero. The KSG estimator performs reliably well when the number of samples of data (which is the number of trials available in our work) is greater than 100, based on testing with simulated data, and available literature. Hence, the patients chosen for this task were those that had atleast 200 trials performed in the picture naming task. Also, the KSG estimator has a negative bias, which was corrected by subtracting the average null-DI estimate from the estimated DI value. In the revised paper, we have added a sentence at the start of the section to explain the cause of the bias, and why the method corrects it.

5) p.8. l.103: from here on, the line numbering is faulty

Authors: We thank the reviewers for pointing this error caused by the presence of equations in the section. We have now fixed the line-numbering in the revision provided.

6) p.9: the section about thresholding should be rewritten for clarity

Authors: We thank the reviewers for this feedback. We realize that we have not clearly defined the terms “density thresholding” and “global thresholding”. We have now revised this section and included the definitions of the terms, and we hope the thresholding section now has improved clarity.

7) p.11 the section about Louvain clustering seems excessively verbose and could be condensed

Authors: We appreciate the feedback, and have greatly condensed the section about Louvain clustering.

8) p.12, l.173: do you mean “any of the 3 language tasks”?

Authors: Yes, we do indeed mean any of the 3 language tasks. We have now changed the sentence “If the current stimulation did not disrupt all 3 language tasks tested individually, then that node-pair was considered to be language negative", and replaced “all” with “any of the” for a better sentence structure.

9) p.14, Figure 4: Please rename the “from” and “to” electrodes, e.g. as “source” and “target” or similar.

Authors: We thank the reviewers for the attention to detail. We have now renamed the “from” and “to” electrodes as “source” and “sink", to improve the clarity of the figure.

10) p.16, Figure 5: While the general idea of this figure is good, having “-256 to 0” below the x-axis in A is confusing

Authors: We thank the reviewers for the suggestion to improve the figure. We have now modified Figure 5A, by labelling the time window as “Time window before articulation, AA: -256 to 0ms", and we hope it improves the clarity of the figure.

11) p.17, l.263: What is the relationship here between DCS and brain resection surgeries?

Authors: DCS is the clinical gold standard to identify language critical areas prior to brain resection surgeries, for improving clinical epilepsy. The resections are guided by the knowledge of such areas, to minimize damage to language regions after surgery. We have added a line in the paper to make this more clear. “DCS informs the neurosurgeon of language critical areas, to estimate the risk, and potential outcome of the brain resection surgeries. “

12) p.23, l. 322-332: are these two possible interpretations necessarily mutually exclusive?

Authors: We thank the reviewers for the excellent point about our discussion on the networks. Previously, we said that we interpret networks, as a group of brain regions that connect to serve a cognitive function, and not as an interpretation from a cognitive perspective (like the difference between the Levelt and the Dell models). Indeed, the two interpretations are not necessarily mutually exclusive, and we realize that our statement was not saying what we intended to say. We have now modified the statement in the discussion, and we provide a simple example to explain what we mean. ``For example, for many decades, Broca’s area (inferior frontal regions) was thought to be primarily responsible for speech articulatory processes. While Broca’s area has now been shown to be involved in other cognitive processes as well, the network view assumes that Broca’s area in conjunction with other brain regions forms a network, and the brain network is responsible for speech articulation. ’. We provide this statement to explain our hypothesis: about whether cognitive functions are being supported by different network states, or by single regions? We hope the new revision of the paper has greater clarity.

13) p.23, l. 332-337: these should be in the same paragraph, and in condensed form.

Authors: The provided revision now does include the background about the hypothesis in the same paragraph, with hopefully greater clarity.

14) p.24, l.386-393: It is not clear how NetDI would influence DCS in practice.

Authors: We agree with the reviewers that the last paragraph of the discussion in the previous version of the paper was not very clear about how NetDI would influence DCS in practice. We have now included a section about future work, and we explain how NetDI could influence DCS. We provide the text here for reference. “In future work, NetDI could be used to improve pre-surgical DCS language mapping in patients. DCS has some unpleasant side-effects, it sometimes induces seizures and has after-discharge effects, so the doctors would prefer to test only as many regions as is essential for clinical mapping. Network and power measures can be found for all nodes, from experiments prior to performing DCS. Given a few ground truth node-pairs, the corresponding network measures could be used as training data to identify other potential language positive and negative areas, to guide doctors in making DCS more clinically efficient.”

15) The results of Figure 6 are not discussed. How does out-degree behave in relation to high gamma, and how to interpret the negative correlation of high-gamma and in-degree?

Authors: We thank the reviewers for these helpful suggestions. Based on this comment, and major comments 4, and 10, we have extended our analysis to include 3 network features (coreness, in-degrees and out-degrees) instead of just in-degrees. Our new figures reflect results from all 3 network features. In the previous version of the paper, we had noted that the number of correlated electrodes were fewer for out-degrees than in-degrees, and hence only showed the results for in-degrees. In this paper, we expanded the analysis to include coreness of nodes, which is a larger scale metric that combines the effects of in-degrees and out-degrees, and more electrodes were correlated with coreness than even in-degrees. We also interpret the negative correlation of in-degrees with high-gamma - as probable evidence that there was an increase in network activity that corresponded to the decrease of high gamma power. This combined with the DCS results indicate that coreness of nodes is a valuable feature of the language process, that has information about the process beyond that which is contained in the power feature.

16) Line 286: “(1) For 3 out of 4 patients, the Gaussian Process Classifier had the highest accuracy, and was thus identified as the best classifier.” Could the authors substantiate this claim, maybe by adding an additional row in Table 3, showing the mean accuracy for each patient and classifier.

Authors: We thank the reviewers for the suggestions. Based on the feedback, in this revision of the paper, we do not limit any result to a single classifier, and instead look at the average performance of all classifiers. Since our goal is to compare feature spaces, we have incorporated the reviewers feedback and have added an additional column in the Table 3, to show the mean accuracy for each feature space.

17) Line 304: Is it the correct that after the Louvain clustering many nodes were not inside any community? i.e., community of size 1, as at initialization? It might then be helpful to show on figure 12A the percentage of nodes that are within a community, to understand how significant the finding is that a lot PN-positive nodes are within a community.

Authors: The reviewers are correct in understanding that many nodes do not belong to any Louvain community. Our observation was that the PN positive node pairs mostly belonged to the Louvain communities during the SA windows, and the maximum number of them were in the communities in SA window 5. The percentage of nodes within a community totally depends on the size of the network, and the number of edges. The proportion of nodes in the network in the communities does correspond to the coarse scale network graph - connection density. More nodes belong to communities during “peaks” of the coarse scale graph. Based on the major comments 6 and 15, we decided to take the reviewer’s feedback and eliminate figure 12 from the paper, and discuss potential implications about the role of PN positive node -pairs in the network as future extensions of the work.

18) Line: 306: Are the authors are claiming that the node-pairs that are PN positive, are usually nodes that are part of 2 different Louvain communities? Can this be quantified?

Authors: We thank the reviewers for their insightful questions, and apologize for not being very clear in this section. We do not claim that the PN positive nodes were part of two different communities, but rather comment that they even showed up as belonging to some community. Belonging to a Louvain community was a property of the algorithm, that identified the community as having greater connectivity within, than with other communities. Many nodes do not belong to any communities. The interesting finding was that the PN nodes were mostly part of communities during the SA windows, and most showed up in window 5, indicating that it could be the potential time when the picture naming task engaged these PN positive regions. However, we agree that more research needs to be done to quantitatively prove that, and we discuss that as future research directions.

## References

[B1] Amblard PO, Michel OJJ (2011) On directed information theory and granger causality graphs. J Comput Neurosci 30:7–16. 10.1007/s10827-010-0231-x 20333542

[B2] Amblard PO, Michel OJ (2012) The relation between granger causality and directed information theory: a review. Entropy 15:113–143. 10.3390/e15010113

[B3] Barnett L, Seth AK (2014) The mvgc multivariate granger causality toolbox: a new approach to granger-causal inference. J Neurosci Methods 223:50–68. 10.1016/j.jneumeth.2013.10.018 24200508

[B4] Barrett AB, Murphy M, Bruno M-A, Noirhomme Q, Boly M, Laureys S, Seth AK (2012) Granger causality analysis of steady-state electroencephalographic signals during propofol-induced anaesthesia. PLoS One 7:e29072. 10.1371/journal.pone.0029072 22242156 PMC3252303

[B5] Bassett DS, Bullmore ET (2009) Human brain networks in health and disease. Curr Opin Neurol 22:340–347. 10.1097/WCO.0b013e32832d93dd 19494774 PMC2902726

[B6] Bassett DS, Porter MA, Wymbs NF, Grafton ST, Carlson JM, Mucha PJ (2013) Robust detection of dynamic community structure in networks. Chaos 23:013142. 10.1063/1.4790830 23556979 PMC3618100

[B7] Bassett DS, Yang M, Wymbs NF, Grafton ST (2015) Learning-induced autonomy of sensorimotor systems. Nat Neurosci 18:744–751. 10.1038/nn.3993 25849989 PMC6368853

[B8] Bentley JL (1975) Multidimensional binary search trees used for associative searching. Commun ACM 18:509–517. 10.1145/361002.361007

[B9] Betzel RF, Bassett DS (2017) Multi-scale brain networks. Neuroimage 160:73–83. 10.1016/j.neuroimage.2016.11.006 27845257 PMC5695236

[B10] Betzel RF, Medaglia JD, Papadopoulos L, Baum GL, Gur R, Gur R, Roalf D, Satterthwaite TD, Bassett DS (2017) The modular organization of human anatomical brain networks: accounting for the cost of wiring. Netw Neurosci 1:42–68. 10.1162/NETN_a_00002 30793069 PMC6372290

[B11] Blank I, Balewski Z, Mahowald K, Fedorenko E (2016) Syntactic processing is distributed across the language system. Neuroimage 127:307–323. 10.1016/j.neuroimage.2015.11.069 26666896 PMC4755877

[B12] Blondel VD, Guillaume J-L, Lambiotte R, Lefebvre E (2008) Fast unfolding of communities in large networks. J Stat Mech 2008:P10008. 10.1088/1742-5468/2008/10/P10008

[B13] Braun U, Schäfer A, Walter H, Erk S, Romanczuk-Seiferth N, Haddad L, Schweiger JI, Grimm O, Heinz A, Tost H, Meyer-Lindenberg A, Bassett DS (2015) Dynamic reconfiguration of frontal brain networks during executive cognition in humans. Proc Natl Acad Sci USA 112:11678–11683. 10.1073/pnas.1422487112 26324898 PMC4577153

[B14] Bressler SL, Seth AK (2011) Wiener–Granger causality: a well established methodology. Neuroimage 58:323–329. 10.1016/j.neuroimage.2010.02.059 20202481

[B15] Bullmore E, Sporns O (2009) Complex brain networks: graph theoretical analysis of structural and functional systems. Nat Rev Neurosci 10:186–198. 10.1038/nrn2575 19190637

[B16] Bullmore E, Sporns O (2012) The economy of brain network organization. Nat Rev Neurosci 13:336–349. 10.1038/nrn3214 22498897

[B17] Chai LR, Mattar MG, Blank IA, Fedorenko E, Bassett DS (2016) Functional network dynamics of the language system. Cereb Cortex 26:4148–4159. 10.1093/cercor/bhw238 27550868 PMC5066829

[B18] Cogan GB, Thesen T, Carlson C, Doyle W, Devinsky O, Pesaran B (2014) Sensory–motor transformations for speech occur bilaterally. Nature 507:94–98. 10.1038/nature12935 24429520 PMC4000028

[B19] Conner CR, Chen G, Pieters TA, Tandon N (2014) Category specific spatial dissociations of parallel processes underlying visual naming. Cereb Cortex 24:2741–2750. 10.1093/cercor/bht130 23696279 PMC4153810

[B20] Crone NE, Boatman D, Gordon B, Hao L (2001) Induced electrocorticographic gamma activity during auditory perception. Clin Neurophysiol 112:565–582. 10.1016/S1388-2457(00)00545-9 11275528

[B21] Domenico MD (2017) Multilayer modeling and analysis of human brain networks. Giga Sci 6:gix004. 10.1093/gigascience/gix004 PMC5437946 28327916

[B22] Dell GS (1988) The retrieval of phonological forms in production: tests of predictions from a connectionist model. J Mem Lang 27:124–142. 10.1016/0749-596X(88)90070-8

[B23] Dell GS, Chang F, Griffin ZM (1999) Connectionist models of language production: lexical access and grammatical encoding. Cogn Sci 23:517–542. 10.1207/s15516709cog2304_6

[B24] Diks C, DeGoede J (2001) A general nonparametric bootstrap test for Granger causality. In: Global analysis of dynamical systems, pp 391–403. Boca Raton: CRC Press.

[B25] Dwyer DB, Harrison BJ, Yücel M, Whittle S, Zalesky A, Pantelis C, Allen NB, Fornito A (2014) Large-scale brain network dynamics supporting adolescent cognitive control. J Neurosci 34:14096–14107. 10.1523/JNEUROSCI.1634-14.2014 25319705 PMC6705292

[B26] Edwards E, Soltani M, Deouell LY, Berger MS, Knight RT (2005) High gamma activity in response to deviant auditory stimuli recorded directly from human cortex. J Neurophysiol 94:4269–4280. 10.1152/jn.00324.2005 16093343

[B27] Fan RE, Chang KW, Hsieh CJ, Wang XR, Lin CJ (2008) Liblinear: a library for large linear classification. J Mach Learn Res 9:1871–1874.

[B28] Fedorenko E, Blank IA (2020) Broca’s area is not a natural kind. Trends Cogn Sci 24:270–284. 10.1016/j.tics.2020.01.001 32160565 PMC7211504

[B29] Fedorenko E, Thompson-Schill SL (2014) Reworking the language network. Trends Cogn Sci 18:120–126. 10.1016/j.tics.2013.12.006 24440115 PMC4091770

[B30] Flinker A, Korzeniewska A, Shestyuk A, Franaszczuk P, Dronkers N, Knight RT, Crone NE (2015) Redefining the role of broca’s area in speech. Proc Natl Acad Sci USA 112:2871–2875. 10.1073/pnas.1414491112 25730850 PMC4352780

[B31] Forseth KJ, Kadipasaoglu CM, Conner CR, Hickok G, Knight RT, Tandon N (2018) A lexical semantic hub for heteromodal naming in middle fusiform gyrus. Brain 141:2112–2126. 10.1093/brain/awy120 29860298 PMC6365955

[B32] Fridriksson J, den Ouden D-B, Hillis AE, Hickok G, Rorden C, Basilakos A, Yourganov G, Bonilha L (2018) Anatomy of aphasia revisited. Brain 141:848–862. 10.1093/brain/awx363 29360947 PMC5837461

[B33] Gao W, Oh S, Viswanath P (2018) Demystifying fixed k-nearest neighbor information estimators. IEEE Trans Inform Theory 64:5629–5661. 10.1109/TIT.2018.2807481

[B34] Geschwind N (1974) Disconnexion syndromes in animals and man. In: Selected papers on language and the brain, pp 105–236. San Diego: Springer.

[B35] Gratton C, Nomura EM, Pérez F, D'Esposito M (2012) Focal brain lesions to critical locations cause widespread disruption of the modular organization of the brain. J Cogn Neurosci 24:1275–1285. 10.1162/jocn_a_00222 22401285 PMC3575518

[B36] Hagmann P, Cammoun L, Gigandet X, Meuli R, Honey CJ, Wedeen VJ, Sporns O (2008) Mapping the structural core of human cerebral cortex. PLoS Biol 6:e159. 10.1371/journal.pbio.0060159 18597554 PMC2443193

[B37] Hagoort P (2014) Nodes and networks in the neural architecture for language: Broca’s region and beyond. Curr Opin Neurobiol 28:136–141. 10.1016/j.conb.2014.07.013 25062474

[B38] Herbet G, Duffau H (2020) Revisiting the functional anatomy of the human brain: toward a meta-networking theory of cerebral functions. Physiol Rev 100:1181–1228. 10.1152/physrev.00033.2019 32078778

[B39] Hickok G, Poeppel D (2007) The cortical organization of speech processing. Nat Rev Neurosci 8:393–402. 10.1038/nrn2113 17431404

[B40] Indefrey P, Levelt WJ (2004) The spatial and temporal signatures of word production components. Cognition 92:101–144. 10.1016/j.cognition.2002.06.001 15037128

[B41] Jiang R, Zuo N, Ford JM, Qi S, Zhi D, Zhuo C, Xu Y, Fu Z, Bustillo J, Turner JA, Calhoun VD, Sui J (2020) Task-induced brain connectivity promotes the detection of individual differences in brain-behavior relationships. Neuroimage 207:116370. 10.1016/j.neuroimage.2019.116370 31751666 PMC7345498

[B42] Kadipasaoglu C, Baboyan V, Conner C, Chen G, Saad Z, Tandon N (2014) Surface-based mixed effects multilevel analysis of grouped human electrocorticography. Neuroimage 101:215–224. 10.1016/j.neuroimage.2014.07.006 25019677

[B43] Kadipasaoglu C, Forseth K, Whaley M, Conner C, Rollo M, Baboyan V, Tandon N (2015) Development of grouped icEEG for the study of cognitive processing. Front Psychol 6:1008. 10.3389/fpsyg.2015.01008 26257673 PMC4508923

[B44] Kadipasaoglu CM, Conner CR, Baboyan VG, Rollo M, Pieters TA, Tandon N (2017) Network dynamics of human face perception. PLoS One 12:e0188834. 10.1371/journal.pone.0188834 29190811 PMC5708727

[B45] Kaplan E, Goodglass H 2nd, Weintraub S (1983) The Boston naming test. Philadelphia: Lea and Febiger.

[B46] Kitsak M, Gallos LK, Havlin S, Liljeros F, Muchnik L, Stanley HE, Makse HA (2010) Identification of influential spreaders in complex networks. Nature Phys 6:888–893. 10.1038/nphys1746

[B47] Kowalik ZJ, Wrobel A, Rydz A (1996) Why does the human brain need to be a nonlinear system? Behav Brain Sci 19:302–303. 10.1017/S0140525X0004276X

[B48] Kramer G (1998) Directed information for channels with feedback. PhD thesis. Zurich: ETH Zurich.

[B49] Kraskov A, Stögbauer H, Grassberger P (2004) Estimating mutual information. Phys Rev E Stat Nonlin Soft Matter Phys 69:066138–066Jun. 10.1103/PhysRevE.69.066138 15244698

[B50] Lam NH, Schoffelen J-M, Uddén J, Hultén A, Hagoort P (2016) Neural activity during sentence processing as reflected in theta, alpha, beta, and gamma oscillations. Neuroimage 142:43–54. 10.1016/j.neuroimage.2016.03.007 26970187

[B51] Lancichinetti A, Fortunato S (2012) Consensus clustering in complex networks. Sci Rep 2:336. 10.1038/srep00336 22468223 PMC3313482

[B52] Levelt WJ (1989) Speaking: from intention to articulation. Cambridge: The MIT Press.

[B53] Lindner M (2011) Trentool. Available at http://trentool.github.io/TRENTOOL3/.

[B54] Lindner M, Vicente R, Priesemann V, Wibral M (2011) Trentool: a MATLAB open source toolbox to analyse information flow in time series data with transfer entropy. BMC Neurosci 12:119. 10.1186/1471-2202-12-119 22098775 PMC3287134

[B55] Liu Y (2012) Directed information for complex network analysis from multivariate time series. East Lansing: Michigan State University. Electrical Engineering.

[B56] Liu Y, Aviyente S (2012) The relationship between transfer entropy and directed information. Proceedings of the IEEE Statistical Signal Processing Workshop (SSP). pp 73–76. Ann Arbor, 5–8 August 2012. 10.1109/SSP.2012.6319809

[B57] Malladi R, Kalamangalam G, Tandon N, Aazhang B (2016) Identifying seizure onset zone from the causal connectivity inferred using directed information. IEEE J Sel Top Signal Process 10:1267–1283. 10.1109/JSTSP.2016.2601485

[B58] Martinet LE, Kramer M, Viles W, Perkins L, Spencer E, Chu C, Cash S, Kolaczyk E (2020) Robust dynamic community detection with applications to human brain functional networks. Nat Commun 11:1–13. 10.1038/s41467-020-16285-7 32503997 PMC7275079

[B59] Massey J (1990) Causality, feedback and directed information. Proc Int Symp Inf Theory Appl (ISITA-90), pp 303–305.

[B60] Medaglia JD, Lynall M-E, Bassett DS (2015) Cognitive network neuroscience. J Cogn Neurosci 27:1471–1491. 10.1162/jocn_a_00810 25803596 PMC4854276

[B61] Modha DS, Singh R (2010) Network architecture of the long-distance pathways in the macaque brain. Proc Natl Acad Sci USA 107:13485–13490. 10.1073/pnas.1008054107 20628011 PMC2922151

[B62] Munding D, Dubarry AS, Alario FX (2016) On the cortical dynamics of word production: a review of the meg evidence. Lang Cogn Neurosci 31:441–462. 10.1080/23273798.2015.1071857

[B63] Murin Y (2017) k-nn estimation of directed information. arXiv:1711.08516.

[B64] Murin Y, Kim J, Goldsmith A (2016) Tracking epileptic seizure activity via information theoretic graphs. In 2016 50th Asilomar Conference on Signals, Systems and Computers, pp 583–587. IEEE.

[B65] Murin Y, Goldsmith A, Aazhang B (2019) Estimating the memory order of electrocorticography recordings. IEEE Trans Biomed Eng 66:2809–2822. 10.1109/TBME.2019.2896076 30714907

[B66] Newman MEJ, Girvan M (2004) Finding and evaluating community structure in networks. Phys Rev E Stat Nonlin Soft Matter Phys 69:e026113. 10.1103/PhysRevE.69.026113 14995526

[B67] Ojemann G, Ojemann J, Lettich E, Berger M (1989) Cortical language localization in left, dominant hemisphere: an electrical stimulation mapping investigation in 117 patients. J Neurosurg 71:316–326. 10.3171/jns.1989.71.3.0316 2769383

[B68] Park PJ, Manjourides J, Bonetti M, Pagano M (2009) A permutation test for determining significance of clusters with applications to spatial and gene expression data. Comput Stat Data Anal 53:4290–4300. 10.1016/j.csda.2009.05.031 21258660 PMC3023458

[B69] Pedregosa F, Varoquaux G, Gramfort A, Michel V, Thirion B, Grisel O, Blondel M, Prettenhofer P, Weiss R, Dubourg V, Vanderplas J, Passos A, Cournapeau D, Brucher M, Perrot M, Duchesnay E (2011) Scikit-learn: machine learning in Python. J Mach Learn Res 12:2825–2830.

[B70] Pei S, Muchnik L, Andrade JS Jr, Zheng Z, Makse HA (2014) Searching for superspreaders of information in real-world social media. Sci Rep 4:5547. 10.1038/srep05547 24989148 PMC4080224

[B71] Price CJ (2010) The anatomy of language: a review of 100 fMRI studies published in 2009. Ann NY Acad Sci 1191:62–88. 10.1111/j.1749-6632.2010.05444.x 20392276

[B72] Quinn CJ, Kiyavash N, Coleman TP (2012) Directed information graphs. CoRR abs/1204.2003.

[B73] Rasmussen CE (2003) Gaussian processes in machine learning. In: Summer school on machine learning, pp 63–71. Berlin: Springer.

[B74] Rasmussen CE, Nickisch H (2010) Gaussian processes for machine learning (gpml) toolbox. J Mach Learn Res 11:3011–3015.

[B75] Reichardt J, Bornholdt S (2006) Statistical mechanics of community detection. Phys Rev E Stat Nonlin Soft Matter Phys 74:016110. 10.1103/PhysRevE.74.016110 16907154

[B76] Riès S, Janssen N, Burle B, Alario FX (2013) Response-locked brain dynamics of word production. PLoS One 8:e58197. 10.1371/journal.pone.0058197 23554876 PMC3595260

[B77] Riès SK, Dhillon RK, Clarke A, King-Stephens D, Laxer KD, Weber PB, Kuperman RA, Auguste KI, Brunner P, Schalk G, Lin JJ, Parvizi J, Crone NE, Dronkers NF, Knight RT (2017) Spatiotemporal dynamics of word retrieval in speech production revealed by cortical high-frequency band activity. Proc Natl Acad Sci USA 114:E4530–E4538. 10.1073/pnas.1620669114 28533406 PMC5468648

[B78] Ronhovde P, Nussinov Z (2009) Multiresolution community detection for megascale networks by information-based replica correlations. Phys Rev E Stat Nonlin Soft Matter Phys 80:016109. 10.1103/PhysRevE.80.016109 19658776

[B79] Rubinov M, Sporns O (2010a) Brain connectivity toolbox. Available at https://sites.google.com/site/bctnet/. 10.1016/j.neuroimage.2009.10.003 19819337

[B80] Rubinov M, Sporns O (2010b) Complex network measures of brain connectivity: uses and interpretations. Neuroimage 52:1059–1069. 10.1016/j.neuroimage.2009.10.003 19819337

[B81] Rubinov M, Sporns O (2011) Weight-conserving characterization of complex functional brain networks. Neuroimage 56:2068–2079. 10.1016/j.neuroimage.2011.03.069 21459148

[B82] Salehi M, Karbasi A, Barron DS, Scheinost D, Constable RT (2020) Individualized functional networks reconfigure with cognitive state. Neuroimage 206:116233. 10.1016/j.neuroimage.2019.116233 31574322 PMC7216521

[B83] Salmelin R, Hari R, Lounasmaa O, Sams M (1994) Dynamics of brain activation during picture naming. Nature 368:463–465. 10.1038/368463a0 8133893

[B84] Saravani AG, Forseth KJ, Tandon N, Pitkow X (2019) Dynamic brain interactions during picture naming. eNeuro 6:ENEURO.0472-18.2019. 10.1523/ENEURO.0472-18.2019 PMC6624411 31196941

[B85] Schreiber T (2000) Measuring information transfer. Phys Rev Lett 85:461–464. 10.1103/PhysRevLett.85.461 10991308

[B86] Seth AK, Barrett AB, Barnett L (2015) Granger causality analysis in neuroscience and neuroimaging. J Neurosci 35:3293–3297. 10.1523/JNEUROSCI.4399-14.2015 25716830 PMC4339347

[B87] Shin K, Eliassi-Rad T, Faloutsos C (2016) Corescope: graph mining using k-core analysis: patterns, anomalies and algorithms, pp 469–478. Piscataway: IEEE.

[B88] Sinai A, Bowers CW, Crainiceanu CM, Boatman D, Gordon B, Lesser RP, Lenz FA, Crone NE (2005) Electrocorticographic high gamma activity versus electrical cortical stimulation mapping of naming. Brain 128:1556–1570. 10.1093/brain/awh491 15817517

[B89] Skeide MA, Friederici AD (2016) The ontogeny of the cortical language network. Nat Rev Neurosci 17:323–332. 10.1038/nrn.2016.23 27040907

[B90] Sporns O (2010) Networks of the brain. Cambridge: The MIT Press.

[B91] Stokes PA, Purdon PL (2017) A study of problems encountered in granger causality analysis from a neuroscience perspective. Proc Natl Acad Sci USA 114:E7063–E7072. 10.1073/pnas.1704663114 28778996 PMC5576801

[B92] Sun Y, Danila B, Josić K, Bassler KE (2009) Improved community structure detection using a modified fine-tuning strategy. Europhys Lett 86:28004. 10.1209/0295-5075/86/28004

[B93] Szelényi A, Joksimovic B, Seifert V (2007) Intraoperative risk of seizures associated with transient direct cortical stimulation in patients with symptomatic epilepsy. J Clin Neurophysiol 24:39–43. 17277576 10.1097/01.wnp.0000237073.70314.f7

[B94] Towle VL, Yoon HA, Castelle M, Edgar JC, Biassou NM, Frim DM, Spire JP, Kohrman MH (2008) Ecog gamma activity during a language task: differentiating expressive and receptive speech areas. Brain 131:2013–2027. 10.1093/brain/awn147 18669510 PMC2724904

[B95] van Wijk BCM, Stam CJ, Daffertshofer A (2010) Comparing brain networks of different size and connectivity density using graph theory. PLoS One 5:e13701. 10.1371/journal.pone.0013701 21060892 PMC2965659

[B96] Varoquaux G, Raamana PR, Engemann DA, Hoyos-Idrobo A, Schwartz Y, Thirion B (2017) Assessing and tuning brain decoders: cross-validation, caveats, and guidelines. Neuroimage 145:166–179. 10.1016/j.neuroimage.2016.10.038 27989847

[B97] Xiong Y, Newman S (2021) Both activation and deactivation of functional networks support increased sentence processing costs. Neuroimage 225:117475. 10.1016/j.neuroimage.2020.117475 33169698

