# Supplementary material for: NetDI: Methodology Elucidating the Role of Power and Dynamical Brain Network Features That Underpin Word Production
Source: eNeuro. 2021 Jan 5;8(1):ENEURO.0177-20.2020. doi: 10.1523/ENEURO.0177-20.2020 (PMC7890525; doi:10.1523/ENEURO.0177-20.2020)
Supplement: Extended Data Table 3-3 — Sensitivity (recall or TPR), specificity (selectivity or TNR), precision for Gaussian process classification Download Table 3-3, ZIP file. [file enu-eN-NWR-0177-20-s04.zip › eNeuro_NetDI_Extended_rev2.pdf]

Extended data

Correlation of in/out degrees with power in various frequency bands: Correlation of in-degrees' and out-degrees' timeseries with the power in 5 different frequency bands was done, and the spearman correlation coefficients are shown in Fig. 6-1. Figure 6-2 depicts the brain regions and number of recording electrodes in each region for all patients that were used in the analysis. The first column of brain ID numbers correspond with the grouping of rows in the Figure. 6-1. The brain regions are broadly grouped into frontal, motor, temporal, occipital and parietal regions of the cortex. An intuitive pictorial representation of the positive and negative correlations are shown in Figure 6-3.

Correlation of three features spaces for all time windows, SA time windows, AA time windows Figure 7-1 is an extension of Figure 7 in the main paper, where the correlation coefficients are calculated for various sets of time-series. It can be noted that most electrodes' feature spaces show correlation in the same direction. There are electrodes where the correlation in SA and AA are in the opposite directions, and are canceled out in the combined SA+AA correlation space. Overall, more electrodes have significant correlation of coreness of nodes feature space with power, than the other two feature spaces, in all three various time-window considerations.

Sensitivity, Specificity and Precision for each classifier: The sensitivity (also called recall or true positive rate TPR), specificity (also called selectivity or true negative rate TNR) and precision for each classifier, patient and feature space are listed below in the Tables 3-1,3-2, 3-3. Each value is shows with it's 95% confidence interval, obtained after 100 re-splits of the data to perform 5-fold cross validation. The results are based on the test segments of the the data partitions, which were all ensured to be balanced. The gray cells denote the highest accuracy obtained across the feature spaces, for that patient. These highest accuracy values are shown in the main paper in Table 3.

k-Nearest Neighbor	Patient 1			Patient 4			Patient 5			Patient 7		
Classifier (KNN)	Sensitivity TPR	Specificity TNR	Precision TP/(TP+FP)	Sensitivity TPR	Specificity TNR	Precision	Sensitivity TPR	Specificity TNR	Precision	Sensitivity TPR	Specificity TNR	Precision
Power	86.6 ± 1.1	63.7 ± 2.6	87.7 ± 0.9	88.1 ± 1.2	0 ± 0	74.5 ± 0.3	95.6 ±1.5	57.3 ± 1.9	49.8 ± 1.6	54.0 ± 2.7	79.8 ± 2.1	71.5 ± 2.9
Indeg	96.7 ± 0.6	35.1 ± 2.5	81.3 ± 0.7	100.0 ± 0	0.3 ± 0.3	77.3 ± 0.1	35.7 ± 2.7	72.5 ± 1.8	35.7 ± 3.0	52.7 ± 2.6	90.1 ± 2.0	80.8 ± 3.0
Outdeg	73.9 ± 1.6	43.7 ± 2.7	79.2 ± 1.0	99.3 ± 0.4	59.6 ± 2.3	89.7 ± 0.6	7.0 ± 1.5	73.0 ± 1.5	5.7 ± 1.4	44.7 ± 2.6	100.0 ± 0	80.8 ± 3.5
Indeg+Outdeg	84.3 ± 1.2	37.4 ± 2.8	79.8 ± 0.9	96.5 ± 0.6	5.3 ± 1.4	77.6 ± 0.3	40.0 ± 2.9	68.0 ± 1.7	31.9 ± 2.6	44.8 ± 2.5	97.0 ± 1.0	79.7 ± 3.3
Cores	79.9 ± 1.3	54.5 ± 2.8	83.9 ± 1.0	90.5 ± 1.0	37.6 ± 2.9	83.6 ± 0.7	55.2 ± 2.7	69.8 ± 1.8	44.6 ± 2.5	55.9 ± 2.8	100.0 ± 0	87.0 ± 2.9
Indeg + Power	88.7 ± 1.1	83.1 ± 2.0	94.0 ± 0.7	82.4 ± 1.1	56.3 ± 2.6	87.0 ± 0.8	76.9 ± 2.8	70.1 ± 1.9	52.2 ± 2.3	43.7 ± 2.4	88.7 ± 1.8	73.4 ± 3.3
Outdeg + Power	74.8 ± 1.4	82.4 ± 2.1	93.1 ± 0.8	89.1 ± 1.2	58.9 ± 2.6	88.5 ± 0.7	87.8 ± 2.4	69.7 ± 1.7	55.2 ± 2.1	54.0 ± 2.6	84.0 ± 2.0	75.4 ± 2.9
In+Outdeg+ Power	86.2 ± 1.2	83.1 ± 2.0	94.1 ± 0.7	82.6 ± 1.3	42.3 ± 2.9	83.6 ± 0.8	76.9 ± 2.6	69.2 ± 1.6	51.6 ± 2.1	51.1 ± 2.7	94.1 ± 1.5	80.7 ± 3.2
Cores + Power	87.9 ± 1.0	77.1 ± 2.5	92.2 ± 0.8	80.5 ± 1.3	47.1 ± 3.1	84.6 ± 0.9	97.3 ± 1.0	66.8 ± 1.8	57.8 ± 1.6	54.4 ± 2.6	89.3 ± 1.8	80.5 ± 2.8

Table 3-1: Sensitivity (recall or TPR), Specificity (selectivity or TNR), Precision for k-Nearest Neighbors classification

Support Vector Machine	Patient 1			Patient 4			Patient 5			Patient 7		
Classifier (SVM)	Sensitivity TPR	Specificity TNR	Precision TP/(TP+FP)	Sensitivity TPR	Specificity TNR	Precision	Sensitivity TPR	Specificity TNR	Precision	Sensitivity TPR	Specificity TNR	Precision
Power	65.3 ± 1.6	52.0 ± 2.5	80.0 ± 1.0	54.5 ± 1.9	8.7 ± 1.7	63.9 ± 1.2	80.0 ± 2.3	39.8 ± 1.6	34.3 ± 1.0	59.4 ± 2.4	73.2 ± 2.8	74.3 ± 2.6
Indeg	52.9 ± 1.6	100.0 ± 0	99.4 ± 0.7	30.1 ± 1.4	79.7 ± 2.3	80.3 ± 2.5	94.1 ± 1.5	34.7 ± 1.9	37.3 ± 0.9	54.9 ± 2.4	100.0 ± 0	91.8 ± 2.4
Outdeg	58.3 ± 1.5	100.0 ± 0	100.0 ± 0	40.9 ± 1.5	100.0 ± 0	98.8 ± 1.0	51.2 ± 3.1	46.0 ± 1.7	25.7 ± 1.5	52.8 ± 2.5	99.9 ± 0.1	88.8 ± 2.8
Indeg+Outdeg	57.7 ± 1.5	100.0 ± 0	100.0 ± 0	39.6 ± 1.5	98.6 ± 0.7	97.3 ± 1.3	89.5 ± 2.2	43.8 ± 1.8	39.1 ± 1.3	54.2 ± 2.5	100.0 ± 0	91.0 ± 2.5
Cores	58.8 ± 1.4	100.0 ± 0	100.0 ± 0	48.4 ± 1.6	82.1 ± 2.1	90.4 ± 1.3	86.6 ± 2.4	42.6 ± 1.8	37.8 ± 1.2	46.5 ± 2.8	87.5 ± 2.0	71.4 ± 3.5
Indeg + Power	59.1 ± 1.8	90.3 ± 1.6	94.3 ± 1.0	48.5 ± 1.9	45.9 ± 2.4	72.8 ± 1.5	81.0 ± 2.3	30.0 ± 1.6	31.1 ± 0.9	50.6 ± 2.4	98.3 ± 1.0	87.8 ± 2.8
Outdeg + Power	58.8 ± 1.7	91.7 ± 1.6	95.2 ± 0.9	40.3 ± 1.6	79.4 ± 2.5	86.5 ± 1.9	93.8 ± 1.8	47.4 ± 1.6	42.2 ± 1.1	56.7 ± 2.5	98.7 ± 0.9	90.4 ± 2.5
In+Outdeg+ Power	57.6 ± 1.4	100.0 ± 0	99.6 ± 0.6	35.0 ± 1.5	79.8 ± 2.4	84.7 ± 2.0	93.5 ± 1.8	42.0 ± 1.6	39.1 ± 1.0	50.2 ± 2.5	99.6 ± 0.5	87.1 ± 2.9
Cores + Power	60.8 ± 1.6	94.8 ± 1.2	97.1 ± 0.7	61.6 ± 1.6	72.9 ± 2.6	88.7 ± 1.1	98.8 ± 0.8	43.6 ± 1.7	42.0 ± 0.9	57.4 ± 2.4	86.9 ± 2.1	82.3 ± 2.6

Table 3-2: Sensitivity (recall or TPR), Specificity (selectivity or TNR), Precision for Support Vector Machine classification

Gaussian Process	Patient 1			Patient 4			Patient 5			Patient 7		
Classifier (GPC)	Sensitivity TPR	Specificity TNR	Precision TP/(TP+FP)	Sensitivity TPR	Specificity TNR	Precision	Sensitivity TPR	Specificity TNR	Precision	Sensitivity TPR	Specificity TNR	Precision
Power	94.0 ± 0.8	43.0 ± 2.9	83.1 ± 0.8	91.0 ± 1.1	2.0 ± 1.0	75.7 ± 0.4	79.5 ± 3.1	62.2 ± 1.9	42.4 ± 1.9	85.8 ± 1.9	82.6 ± 2.7	88.7 ± 1.7
Indeg	95.4 ± 0.7	51.0 ± 3.0	85.4 ± 0.9	98.7 ± 0.4	5.0 ± 1.4	78.0 ± 0.3	0.5 ± 0.4	98.9 ± 0.6	0.3 ± 0.3	71.8 ± 2.8	68.9 ± 4.0	79.0 ± 2.6
Outdeg	87.1 ± 1.3	45.1 ± 3.4	82.9 ± 1.1	98.0 ± 0.7	0.4 ± 0.5	76.8 ± 0.2	2.9 ± 1.1	95.0 ± 1.0	3.9 ± 1.5	55.0 ± 2.5	85.5 ± 1.9	77.7 ± 2.8
Indeg+Outdeg	97.8 ± 0.7	28.6 ± 3.4	80.3 ± 1.0	97.4 ± 0.7	7.2 ± 1.9	78.3 ± 0.4	61.2 ± 2.7	68.8 ± 1.6	44.8 ± 2.2	68.8 ± 2.5	84.2 ± 2.3	84.1 ± 2.3
Cores	91.5 ± 1.1	20.1 ± 2.7	77.1 ± 0.8	94.2 ± 0.8	49.8 ± 3.4	87.4 ± 0.8	62.3 ± 2.9	74.8 ± 1.6	52.5 ± 2.7	74.4 ± 2.7	71.7 ± 3.9	82.7 ± 2.4
Indeg + Power	86.4 ± 1.1	59.1 ± 2.8	86.7 ± 0.9	94.0 ± 1.0	36.3 ± 3.2	84.1 ± 0.8	65.2 ± 2.7	62.9 ± 1.8	42.5 ± 2.0	56.7 ± 2.6	83.1 ± 2.3	76.7 ± 2.9
Outdeg + Power	90.5 ± 1.1	32.3 ± 3.2	80.7 ± 0.9	91.1 ± 1.0	52.8 ± 3.5	87.8 ± 0.9	96.4 ± 1.3	75.2 ± 1.5	64.0 ± 1.8	57.9 ± 2.4	97.3 ± 1.2	90.3 ± 2.5
In+Outdeg+ Power	86.2 ± 1.1	57.9 ± 2.8	86.3 ± 0.9	90.4 ± 1.1	57.1 ± 2.7	88.2 ± 0.7	96.6 ± 1.1	74.5 ± 1.4	63.6 ± 1.7	58.7 ± 2.5	97.1 ± 1.0	90.1 ± 2.4
Cores + Power	91.9 ± 0.9	59.9 ± 2.7	87.5 ± 0.8	96.8 ± 0.8	76.2 ± 2.5	93.8 ± 0.7	81.5 ± 2.3	71.0 ± 1.6	56.0 ± 2.0	69.3 ± 2.1	98.0 ± 1.0	97.1 ± 1.3

Table 3-3: Sensitivity (recall or TPR), Specificity (selectivity or TNR), Precision for Gaussian Process classification

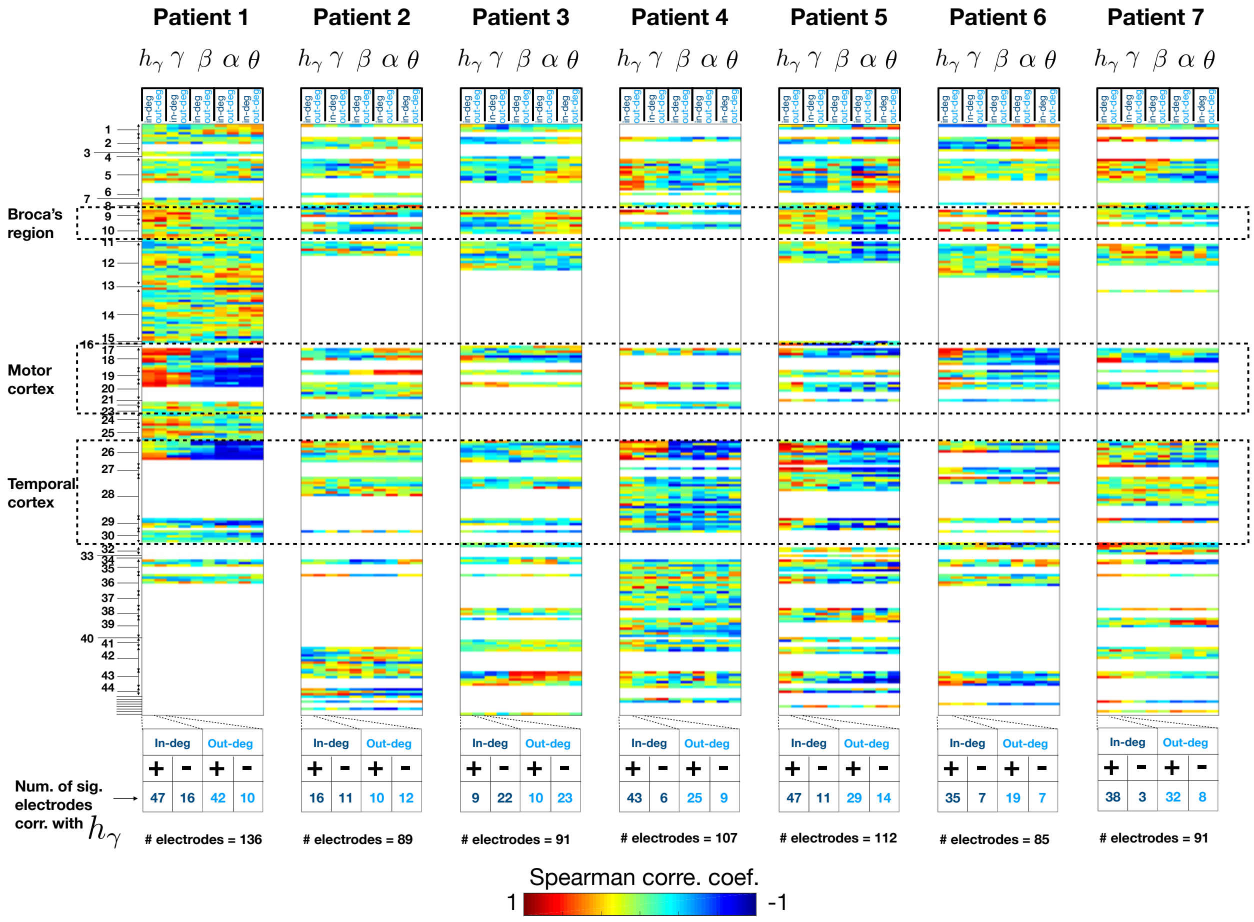

Figure 6-1: A matrix representation of correlation coefficients of in and out degrees with powers in 5 frequency bands are shown for each of the 7 patients. Rows of all matrices are electrodes grouped by brain region, and there are 10 columns for each patient. First 2 columns are corr. coeff. of in-degrees and out-degrees with $h\gamma$ power (60-200Hz), next 2 columns are in/out-degrees with γ power (30-60Hz), next 2 columns are corr. coeff. of in/out-degrees β power (13-30Hz), next 2 columns are corr. coeff. of in/out-degrees α power (8-13Hz), while the last 2 columns are with θ power (4-8Hz). It can be observed that while the electrode coverage of all the patients is different, there exist similar trends of high positive correlation with $h\gamma$ power and negative correlation with the θ power, particularly in language related brain regions. The brain regions are labeled with numbers, and are enumerated in Figure 6-2. The exact number of electrodes that were positively and negatively correlated for in-degrees and out-degrees are also shown, and more electrodes were found to be negatively correlated with out-degrees than in-degrees, while greater number of electrodes are positively correlated with in-degrees than out-degrees.

ID	Brain region name	Pt.1	Pt.2	Pt.3	Pt.4	Pt.5	Pt.6	Pt.7
1	Gyr. rectus	5	1	3	0	2	1	2
2	Sul. orbital-H shaped	3	5	3	2	3	6	2
3	Sul. suborbital	2	0	0	0	0	0	0
4	Sul. orbital lateral	0	0	1	0	1	0	0
5	Gyr. orbital	10	8	10	13	14	9	10
6	Gyr. frontal inf-Orbital part	0	2	0	1	0	0	0
7	Gyr. & Sul. trans. frontopolar	2	0	0	0	0	0	0
8	Sul. frontal inferior	3	3	0	2	3	1	3
9	Gyr. frontal inf-Opercular part	5	3	5	2	5	3	4
10	Gyr. frontal inf-Triangular part	8	5	5	3	5	4	2
11	Sul. frontal middle	1	1	1	0	1	0	0
12	Gyr. frontal middle	17	5	11	0	8	14	9
13	Sul. frontal superior	2	0	0	0	0	0	0
14	Gyr. frontal superior	21	0	0	0	0	0	1
15	Gyr. frontomarginal	1	0	0	0	1	0	0
16	Sul. precentral-Sup.-part	0	0	0	0	1	0	0
17	Sul. precentral-Inf.-part	0	0	1	0	0	0	0
18	Gyr. precentral	9	5	6	3	4	7	6
19	Gyr. subcentral	5	2	2	0	3	4	1
20	Gyr. postcentral	2	7	2	3	4	3	3
21	Sul. postcentral	0	0	0	0	1	1	0
22	Gyr. precuneus	3	0	0	3	0	0	0
23	Gyr. paracentral	2	0	0	0	0	0	0
24	Gyr. cingulate-Main part	5	2	0	0	0	0	0
25	Sul. cingulate-Main part & Intraci	6	0	0	0	0	0	0
26	Gyr. temp sup-Lateral aspect	8	9	9	8	10	5	11
27	Sul. temporal superior	0	0	0	1	4	3	0
28	Gyr. temporal middle	0	8	5	17	6	3	12
29	Gyr. temporal inferior	4	0	3	5	5	2	2
30	Sul. temporal inferior	5	1	1	1	0	1	0
31	Gyr. temp sup-Planum tempolare	0	0	2	0	0	2	2
32	Pole temporal	0	0	0	0	2	1	3
33	Pole occipital	0	0	0	0	1	0	0
34	Sul. occipito-temporal medial	0	0	1	0	0	0	0
35	Gyr. occipit-temp lat-Or fusiform	3	2	2	6	5	3	2
36	Gyr. occipit-temp med-Lingual part	4	1	1	8	4	5	1
37	Gyr. & Sul. occipital inferior	0	0	0	6	0	0	0
38	Gyr. occipit-temp med-Parahippo.	0	0	3	1	4	0	1
39	Gyr. occipital middle	0	0	1	8	2	0	4
40	Gyr. occipital superior	0	0	0	0	1	0	0
41	Gyr. cuneus	0	0	3	3	1	0	0
42	Gyr. parietal inferior-Angular part	0	10	2	2	3	0	5
43	Gyr. parietal inferior-Supramarginal	0	3	6	7	5	6	3
44	Gyr. parietal superior	0	3	0	0	2	0	0
45	Sul. intraparietal-& Parietal tran	0	1	0	0	0	0	0
46	Sul. collateral transverse post	0	0	0	1	0	0	0
47	Sul. collateral transverse ant	0	1	0	1	0	0	1
48	Sul. circular insula inferior	0	0	0	0	0	1	0
49	Gyr. insular short	0	0	0	0	1	0	0
50	Sul. calcarine	0	1	0	0	0	0	0
51	S intermedius primus-Jensen	0	0	0	0	0	0	1
52	Medial wall	0	0	1	0	0	0	0

Figure 6-2: Table depicting the brain regions and number of recording electrodes in each region for all patients

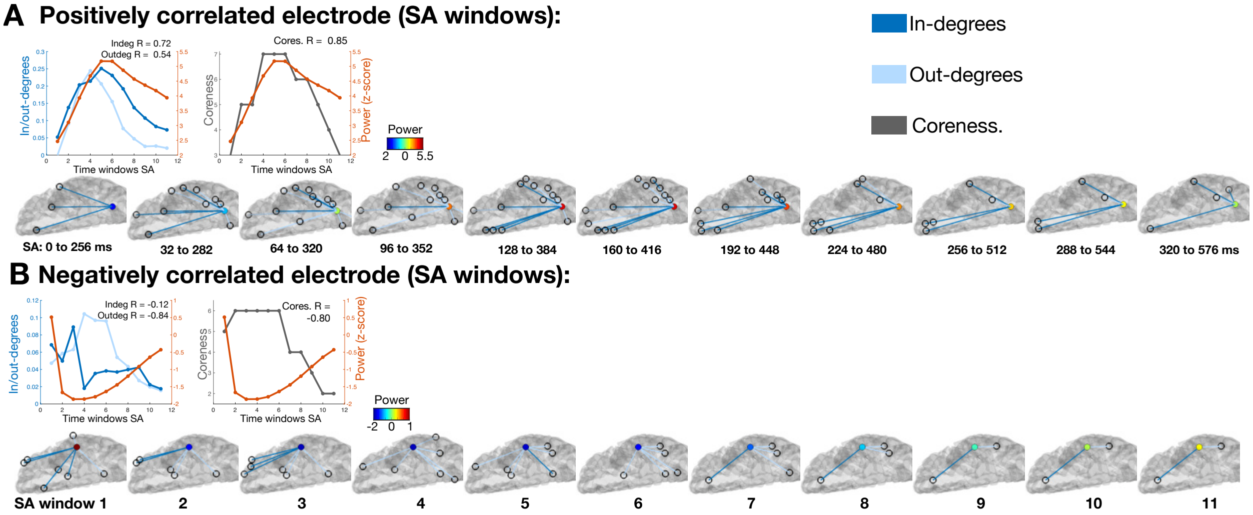

Figure 6-3: (A) A positively correlated occipital-temporal-lateral-fusiform-gyrus electrode from patient 5; (B)A negatively correlated Inf-temporal electrode from patient 5. The colors of the nodes in A-B represent the power in the window, while the dark blue lines are in-degrees to the node, and the light blue lines are the out-degrees. These figures demonstrate that coreness of nodes is due to the combined effect of in and out degrees, and provide a pictorial understanding of how in/out degrees correlates with power.

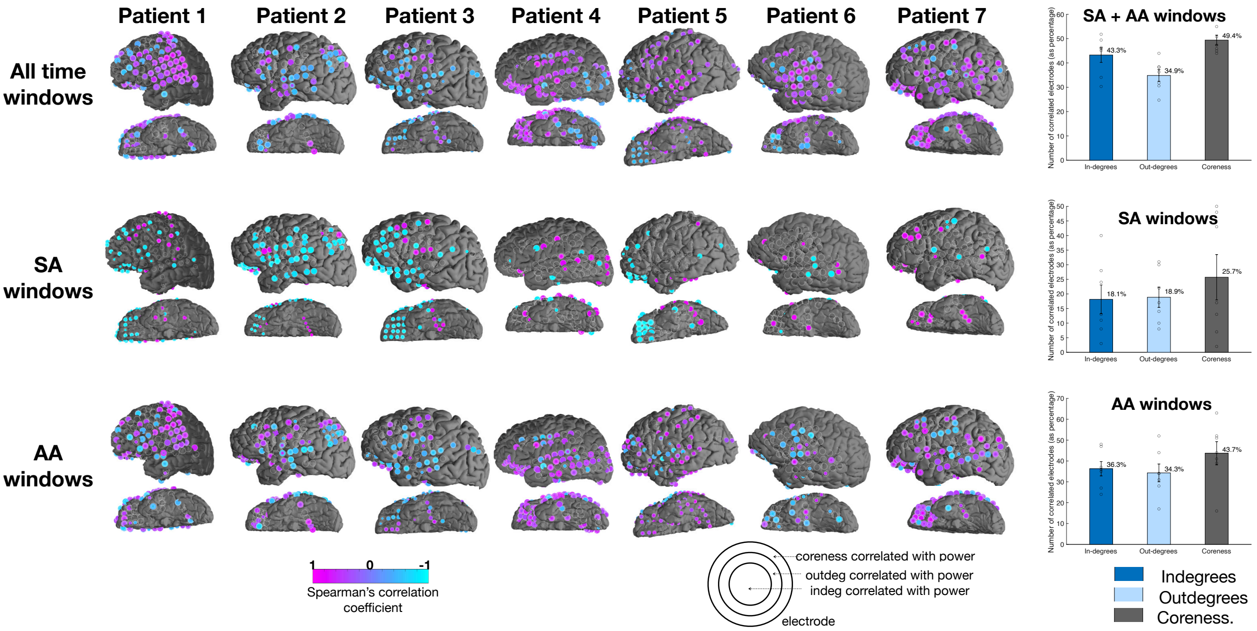

Figure 7-1: Every 3-ring electrode on the brain denotes the correlation coefficient value of three feature spaces with power. The significant corr. coeff. of in-degrees and h_γ power time-series are shown in the innermost circle, the corr. coeff of out-degrees with power is denoted by the color of the middle ring, while the outer ring for each electrode's color denotes the corr. coeff. of coreness of nodes with power. The absence of color in the outer ring, or the absence of either the middle or inner ring denotes the lack of significant correlation in that electrode, with that feature space. The first row denotes the corr. coeff. calculated using the entire time-series, and is the same as the main figure in the paper. The second row shows the corr. coeff. of the three feature spaces with power, when only the SA time windows are considered, while the third row shows the corr. coeff. when only the AA windows are considered. The bar plots to the right denote the average percentage of electrodes that showed significant correlation for each feature space, after correcting for multiple comparisons (FDR, p-val < 0.05 for each feature space, per patient). It can be noted that most electrodes' feature spaces show correlation in the same direction. There are electrodes where the correlation in SA and AA are in the opposite directions, and are canceled out in the combined SA+AA correlation space. Overall, more electrodes have significant correlation of coreness of nodes feature space with power, than the other two feature spaces, in all three various time-window considerations.
